# Obesity and Metabolic Disease Impair the Anabolic Response to Protein Supplementation and Resistance Exercise: A Retrospective Analysis of a Randomized Clinical Trial with Implications for Aging, Sarcopenic Obesity, and Weight Management

**DOI:** 10.3390/nu16244407

**Published:** 2024-12-23

**Authors:** Mats I. Nilsson, Donald Xhuti, Nicoletta Maria de Maat, Bart P. Hettinga, Mark A. Tarnopolsky

**Affiliations:** 1 Exerkine Corporation, McMaster University Medical Center, Hamilton, ON L8N 3Z5, Canada; bart.hettinga@exerkine.com; 2 Department of Pediatrics, McMaster University Medical Center, Hamilton, ON L8N 3Z5, Canada; xhutid@mcmaster.ca (D.X.); nicoletta.demaat@exerkine.com (N.M.d.M.)

**Keywords:** obesity, aging, sarcopenia, sarcopenic obesity, metabolic syndrome, anabolic resistance, exercise, protein, whey, creatine, collagen, vitamin D, omega-3, calcium, weight management, GLP-1, Ozempic

## Abstract

Background: Anabolic resistance accelerates muscle loss in aging and obesity, thus predisposing to sarcopenic obesity. Methods: In this retrospective analysis of a randomized clinical trial, we examined baseline predictors of the adaptive response to three months of home-based resistance exercise, daily physical activity, and protein-based, multi-ingredient supplementation (MIS) in a cohort of free-living, older males (*n* = 32). Results: Multiple linear regression analyses revealed that obesity and a Global Risk Index for metabolic syndrome (MetS) were the strongest predictors of Δ% gains in lean mass (TLM and ASM), LM/body fat ratios (TLM/%BF, ASM/FM, and ASM/%BF), and allometric LM (ASMI, TLM/BW, TLM/BMI, ASM/BW), with moderately strong, negative correlations to the adaptive response to polytherapy r = −0.36 to −0.68 (*p* < 0.05). Kidney function, PA level, and chronological age were only weakly associated with treatment outcomes (*p* > 0.05). Next, we performed a subgroup analysis in overweight/obese participants with at least one other MetS risk factor and examined their adaptive response to polytherapy with two types of protein-based MIS (PLA; collagen peptides and safflower oil, *n* = 8, M5; whey/casein, creatine, calcium, vitamin D_3_, and fish oil, *n* = 12). The M5 group showed greater improvements in LM (ASM; +2% vs. −0.8%), LM/body fat ratios (ASM/FM; +3.8% vs. −5.1%), allometric LM (ASM/BMI; +1.2% vs. −2.5%), strength (leg press; +17% vs. −1.4%), and performance (4-Step-Stair-Climb time; −10.5% vs. +1.1%) vs. the PLA group (*p* < 0.05). Bone turnover markers, indicative of bone accretion, were increased pre-to-post intervention in the M5 group only (P1NP; *p* = 0.036, P1NP/CTX ratio; *p* = 0.088). The overall anabolic response, as indicated by ranking low-to-high responders for Δ% LM (*p* = 0.0079), strength (*p* = 0.097), and performance (*p* = 0.19), was therefore significantly higher in the M5 vs. PLA group (*p* = 0.013). Conclusions: Our findings confirm that obesity/MetS is a key driver of anabolic resistance in old age and that a high-quality, whey/casein-based MIS is more effective than a collagen-based alternative for maintaining musculoskeletal health in individuals at risk for sarcopenic obesity, even when total daily protein intake exceeds current treatment guidelines.

## 1. Introduction

Anabolic resistance of skeletal muscle (SM) is a key driver of muscle loss and dysfunction with aging, disuse, physical inactivity, catabolic disease states, and obesity [1]. Considering the rapid growth of the world’s aging population and soaring rates of obesity and sedentarism, it is essential to understand the complex physiologic and mechanistic basis of anabolic resistance and muscle deterioration in these conditions to develop targeted and effective interventions.

Both intrinsic and extrinsic mechanisms can drive anabolic resistance and impair SM basal protein turnover and the adaptive response to anabolic stimuli (i.e., growth factors, hormones, amino acids, and/or contractile activity), thereby accelerating muscle deterioration and dysfunction. Specifically, the biological aging process (i.e., hallmarks of aging [2]) and acquired risk factors in obesity/metabolic syndrome (MetS), such as excess body fat (BF), dyslipidemia, and dysglycemia, independently fuel a ‘vicious cycle’ of organellar damage, oxidative stress, and inflammation [3], which exacerbates peripheral insulin resistance, adipose tissue expansion, and ectopic lipid deposition (i.e., hepato- and myosteatosis). Furthermore, these independent pathoetiologies may converge into polymorbid conditions, such as sarcopenic obesity, which poses a major health risk for older adults and complicates both diagnosis and disease management [4].

Current management strategies for obesity and MetS broadly focus on either decreasing energy intake, increasing energy output or a combination of both. The first line of treatment is lifestyle management (e.g., daily physical activity (PA), structured exercise, and caloric restriction), while more radical solutions may include weight loss drugs, anti-diabetes medications, and/or bariatric surgery. Unfortunately, only 20% of obese individuals can preserve and stabilize weight loss in the long term, and more than half regain most of their weight loss within a year [5]. Two significant reasons for this are that weight loss is associated with an increased drive to eat and a reduction in resting and non-resting energy expenditure per kilogram of fat-free mass (FFM; water, organs, muscle, and bone) [6,7]. These changes predispose to significant weight regain and cycling [5], which are associated with progressive loss of lean body mass (LBM/LM), an increase in fat mass (FM), and a reduction in LM/FM ratios [8,9].

Based upon impressive weight loss outcomes (~−15 to −20% in the first year), the most popular weight loss methods that are currently being employed are glucagon-like peptide-1 receptor agonists (GLP-1RA; i.e., semaglutide) [10] and dual agonists of both GLP-1 and glucose-dependent insulinotropic polypeptide (GIP; i.e., tirzepatide) [11]. An increasingly recognized drawback of such medications is that ~20–40% of the total weight loss is derived from FFM, with the remainder being FM [12,13,14]. The magnitude of FFM loss is typically governed by the total reduction in body weight, making pharmacotherapy and bariatric surgery high-risk strategies in terms of maintaining musculoskeletal health, with both methods causing substantial SM loss, bone degradation, and a reduction in resting metabolic rate (RMR) [12,15,16]. Excessive muscle deterioration is obviously undesirable for all patients but may be more detrimental for older adults who are predisposed to sarcopenic obesity and SM anabolic resistance, potentially blunting the adaptive response to anabolic therapies and limiting FFM regain following weight loss [1,4]. At the same time, studies have confirmed that weight-bearing exercise and strength training attenuate SM loss [17], and the development and implementation of other low-risk therapies, besides exercise, that mitigate musculoskeletal deterioration are necessary to improve overall safety, efficacy, and acceptability of contemporary weight management programs.

Current strategies for treating sarcopenic obesity are centered around multimodal interventions with the aim of inducing a net negative energy balance for reducing FM, ectopic lipid deposition, and inflammation, with a concurrent increase in muscle mass and strength [4]. These interventions typically include hypocaloric diets (−200–700 kcal/day), increased protein intake (1.2–1.5 g/kg BW/day), and combined aerobic and resistance exercise [4,18], thus significantly overlapping with multimodal interventions for both sarcopenia [19] and obesity [20,21]. In a recent topical review, Prado and colleagues stressed the importance of consuming high-quality proteins that are rich in essential amino acids (i.e., animal > plant-based) for inducing satiety and anabolism and advocated for concurrent intake of polyunsaturated fatty acids (i.e., C20:5*n*-3 and C22:6*n*-3 PUFAs; EPA and DHA), calcium, vitamin D, and antioxidants for targeting other aspects of the underlying etiology (e.g., oxidative stress and inflammation) [4]. While the premise of multi-ingredient supplementation (MIS) for targeting multiple pathways in sarcopenia and obesity is not new [22,23], more research is needed to confirm the importance of high-quality, protein-based MIS for LBM preservation in polymorbid conditions, such as sarcopenic obesity.

In this retrospective analysis of a three-month interventional RCT (Nilsson & Tarnopolsky [22]), we aimed to identify predictors of the adaptive response to home-based resistance exercise (HBRE; 3 days/week), daily physical activity (PA; walking), and protein-based MIS (Placebo; collagen peptides + safflower oil or Muscle 5; whey/casein + creatine + vitamin D_3_ + calcium + fish oil) in a cohort of free-living, older males, including a subgroup of obese, polymorbid individuals at risk for sarcopenic obesity (obese/MetS). Accordingly, we conducted backward stepwise regression on potential predictors of the adaptive response (e.g., baseline age, PA level, protein intake, kidney function, obesity, and MetS risk factors) and proceeded with an independent analysis in the obese/MetS subgroup to determine if the quality of protein-based MIS affected the adaptive response to the multimodal intervention. We hypothesized that obesity and other MetS risk factors would be negatively correlated to the treatment response (i.e., Δ% gains in LBM, strength, and performance) and that MIS with an incomplete protein source (i.e., collagen peptides) would be inferior to a higher-quality alternative (i.e., whey/casein) for mitigating sarcopenic obesity risk, even when daily protein intake meets or exceeds current treatment guidelines.

## 2. Materials and Methods

### 2.1. Ethics, Clinical Trial Registration, and Funding

This is a retrospective analysis of a double-blind, placebo-controlled, randomized clinical trial conducted at the McMaster University Medical Centre (MUMC, Hamilton, ON) between June 2018 and April 2019 [22]. The deidentified and locked data set was re-analyzed between 1 October 2023 and 1 October 2024, and the results herein have not been published previously.

All methods and procedures were approved by the Hamilton Integrated Research Ethics Board (2018-4656-GRA; approval date: 12 June 2018), and the trial was registered at clinicaltrials.gov (NCT03536871).

The funding for the multimodal intervention was provided by a CIHR grant awarded to Dr. Tarnopolsky (#143325). Some of the baseline samples were sent for use in an aging study funded by Buck Institute 2017-0471/Astellas Pharmaceuticals [24].

### 2.2. Consort Flow Chart, Participants, and Sample Size

As previously described [22], this 12-week trial was designed to assess the potential benefits of an exercise- and supplement-based, multimodal intervention under free-living conditions in older males representative of the North American aging community in general (i.e., sedentary/low active, overweight or obese, and with varying degrees of polymorbidity [25,26,27,28]).

General inclusion criteria were ≥65 years of age, male, sedentary or low active (≤150 min of PA per week), and of normal to class 1 obesity body mass index (BMI; 20–34.9). Individuals with class 2 and class 3 obesity were accepted into the study if fulfilling other criteria and were deemed safe for participation. For a detailed list of specific exclusion criteria, please refer to the original publication [22].

**Figure 1:**
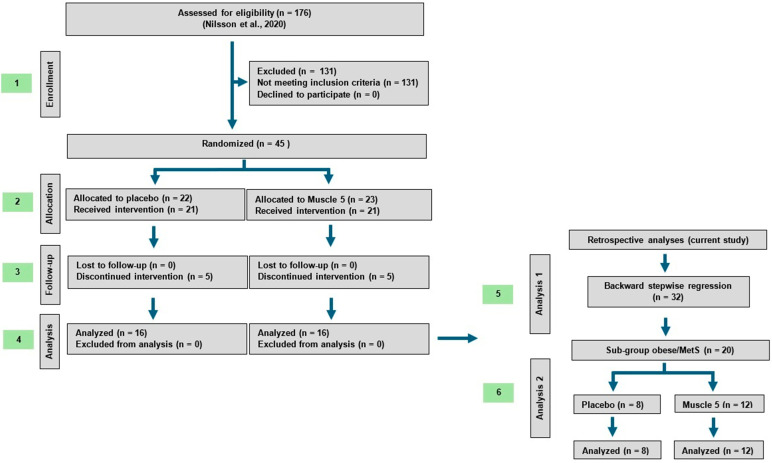
Consort flow chart. Left panel (1–4): Enrollment, allocation, follow-up, and analysis of the original clinical trial (Nilsson et al. [22]). Right panel (5–6): The retrospective data analyses conducted for the current manuscript.

Between June 2018 and January 2019, 179 individuals from the general population in the Greater Hamilton Area were screened for participation (Figure 1). All volunteers were informed about the purpose of the research, the experimental procedures, and potential risks prior to providing their written informed consent for participation in the study. Forty-five individuals were then randomized into two intervention groups, with 32 participants completing pre- and post-clinical testing (Placebo/PLA; *n* = 16, Muscle 5/M5; *n* = 16). Withdrawals and dropouts were in line with other RCTs on unsupervised exercise and/or supplementation with limited subject interaction [29]. Specifically, three individuals withdrew prior to the start of the study, while ten dropped out during the intervention, with an even distribution across groups. Reasons for withdrawal/dropout included unrelated surgery (*n* = 2), unrelated health issues (*n* = 2), muscle soreness (*n* = 1), travel (*n* = 2), flavor of oil (*n* = 1), time constraints (*n* = 1), and other personal reasons (*n* = 4).

The original sample size calculations were based on a clinical trial on the effects of creatine supplementation and resistance exercise (RE) on muscle integrity in older adults (i.e., *n* = 8–11 per group) [30], setting power to 80% and alpha at 5%, yielding an estimate of 10 participants per group for detecting significant differences in body composition and strength. Previously, an authoritative meta-analysis by Morton et al. demonstrated that the average sample size in protein supplementation and RE studies is *n* = 19 [31]. Thus, accounting for an estimated 20–30% dropout rate for unsupervised training forms with limited subject interactions [29], we aimed for a final count of 15–20 participants per group [22].

For the current manuscript, the retrospective analyses were conducted on the full sample size (regression analyses; *n* = 32), followed by a subgroup analysis in obese/MetS individuals with body mass indices ≥27 plus one other risk factor for metabolic syndrome (*n* = 20) (Figure 1).

### 2.3. Study Design and Clinical Testing

**Figure 2:**
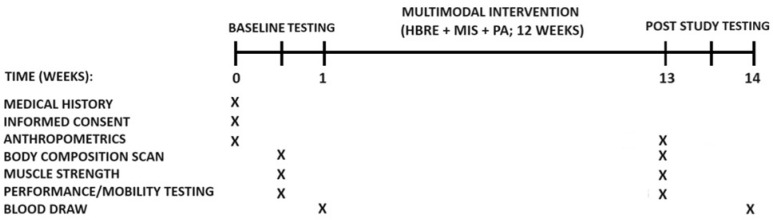
RCT timeline and clinical testing. X = time point of procedure (weeks 0–1 and 13–14).

Briefly, this 12-week multimodal intervention was designed to mimic a ‘real life situation’ in which the participants engaged in unsupervised HBRE (elastic band training, 3 d/week), daily PA (walking), and consumed either a higher- (M5; whey and casein + creatine + vitamin D_3_ + fish oil, *n* = 12) or a lower-quality (PLA; collagen peptides + safflower oil, *n* = 8), protein-based MIS daily. Baseline and post-study clinical tests were identical and consisted of vital signs, anthropometry, body composition, strength, and performance testing (Figure 2). Serum and plasma samples were obtained from venous blood draws to assess kidney and liver function, bone turnover markers, and MetS risk factors. Subjects arrived at the clinic in the fasted state (10–12 h) at the same time (AM) for all testing occasions.

### 2.4. Home-Based Resistance Exercise (HBRE)

As previously described [22], the HBRE program consisted of six upper-body and six lower-body resistance exercises, specifically, biceps curl, triceps extension, lateral raise, seated row, bench press, abdominal crunch, calf raises, chair squat, knee extension, knee flexion, hip flexion, and dorsi flexion. The program followed ACSM guidelines for strength training for older adults (i.e., full-body training 3 days/week on non-consecutive days, 3 × 10–15 repetitions per exercise, and progressive increases in exercise intensity) [32,33], with elastic band tension force (i.e., resistance) progressively increased throughout the study for continual adaptation (yellow, 1.32 kg; red, 1.77 kg; green, 2.27 kg; blue, 3.22 kg; and black, 4.40 kg). Compliance and elastic band resistance were recorded in an exercise log, which was collected post-intervention.

### 2.5. Physical Activity (PA)

Subjects were encouraged to maintain 10,000 steps on non-HBRE days and 5000 steps on HBRE days (i.e., 55,000 steps per week; 7857 steps per day) to improve health-related QoL and obesity/MetS status [34]. Daily PA levels were estimated by step counts (Omron HJ-321 Alvita Pedometer; Omron, Kyoto, Japan) and recorded in a step log.

### 2.6. Multi-Ingredient Supplementation (MIS), Randomization, and Blinding

Following enrollment, participants were coded/deidentified by the investigators and thereafter randomly assigned to the intervention by blocked randomization with an allocation ratio of 1:1 and block size of 4–8 participants by a third party. The allocation was not revealed to the investigators until pre- and post-intervention data had been cross-referenced, locked, and provided to an independent data safety and monitoring committee.

All supplements were manufactured by Gruppo Nutrition (Windsor, ON, Canada) and delivered to the subjects in a double-blind fashion with a coded alphanumerical system. Placebo (PLA) and active (M5) were isocaloric, matched for total protein, and identical in flavor, smell, and appearance. The PLA group received collagen peptides (40 g/d, Peptiplus^®^, Gelita, Eberbach, Germany) and safflower oil (two tsp./d), while the M5 group received two complete protein sources designed to mimic human breast milk (“humanized milk ratio”; 24 g/d whey and 16 g/d casein, i.e., 60:40 ratio), creatine monohydrate (3 g/d), vitamin D_3_ (1000 IU/d), calcium (416 mg/d as calcium caseinate), and fish oil (two tsp./d, EPA; 1.51 g/d, DHA; 0.95 g/d) (Muscle 5^®^ and Omega 3^®^, Stayabove Nutrition, Hamilton, ON, Canada). Because M5 and PLA contained either complete or incomplete protein sources, they may be described as “higher-quality” and “lower-quality” protein-based MIS, respectively. Collagen lacks tryptophan and cysteine and has low levels of methionine, essential amino acids (EAAs) and branched-chain amino acids (BCAAs); thus, a Digestible Indispensable Amino Acid Score (DIAAS) of 0 and significantly lower anabolic potential vs. milk proteins [35,36,37]. For this study, PLA and M5 were matched for total protein (40 g) and contained 15.9% vs. 46.2% EAAs, 6.5% vs. 22.2% BCAAs, and 2.7% vs. 11.4% leucine, respectively (Table S5). All participants were instructed to consume the supplements in the morning with breakfast and to record their daily intake in a supplement log, which was collected at the end of the trial.

### 2.7. Dietary Records

To assess involuntary compensatory mechanisms from exercise participation and/or multi-ingredient supplementation (e.g., increased or decreased drive to eat), the participants were asked to maintain their normal food intake and kept a detailed dietary record for three non-consecutive days at the start and end of the trial, including two weekdays and one day of the weekend. Based on these 72-h recalls, an estimate of pre-to-post changes in energy and macronutrient intakes were obtained for each participant using the ASA-24 software NIH 2018a (National Cancer Institute, Rockville, MD, USA).

### 2.8. Anthropometry and Vital Measures

Basic anthropometry and vital measures were assessed pre- and post-intervention, including body weight, height, body mass index (BMI; kg/m^2^), heart rate, and arterial blood pressure.

### 2.9. Body Composition, Strength, and Performance Tests

A comprehensive test battery consisting of body composition, strength, and performance testing was completed pre- and post-intervention and is considered the reference standard for diagnosing, tracking disease progression, and assessing interventional efficacy in sarcopenia/sarcopenic obesity research [38,39,40,41].

Body composition was assessed by dual-energy X-ray absorptiometry (DXA; GE Lunar Prodigy, Madison, WI, USA), allowing for estimates of bone, lean and fat mass changes pre-to-post intervention. Primary outcomes were total and appendicular lean mass (TLM and ASM), muscle-to-body fat ratios (TLM/FM, TLM/%BF, ASM/FM, and ASM/%BF) and diagnostic criteria related to sarcopenia and sarcopenic obesity (i.e., allometric lean mass; ASMI, TLM/BW, TLM/BMI, ASM/BW, and ASM/BMI). Quality assurance testing with a QA-block was performed in the morning and as needed to ensure machine calibration. All subjects were scanned in the fasted state with body positions, bony landmarks, scan table references, and ROIs kept constant between pre- and post-scans.

Improvements in upper and lower body strength were assessed by 1-RM grip strength, 1-RM leg press, and 1-RM isometric leg extension (secondary outcomes). The muscle quality index (MQ), which is an indicator of strength per unit muscle [42], was then calculated by dividing upper and lower body strength (hand grip + ([leg press + leg extension]/2)) by appendicular lean mass (ASM).

Pre-to-post intervention changes in performance/functional mobility were tracked by the Short Physical Performance Battery (SPPB; 4-m gait speed, 5X sit-to-stand time (5XSTS), and balance testing) and an independent functional mobility test (4-step stair climb; 4SSC) (secondary outcomes).

### 2.10. Blood Collection and Analyses

Blood chemistry was performed on serum or plasma samples obtained from venous blood draws pre- and post-intervention. Diagnostic markers of metabolic syndrome (dyslipidemia, dysglycemia and systemic inflammation) and organ function (kidney and liver) were measured, including creatinine, bilirubin, alanine aminotransferase (ALT), gamma-glutamyltransferase (GGT), C-reactive protein (CRP), low-density lipoprotein (LDL), high-density lipoprotein (HDL), total cholesterol, triglycerides and glycosylated hemoglobin A1c (HbA1c) (Core Laboratory, Health Sciences Centre, Hamilton, UK).

Given the need for the development of adjunctive therapies that can maintain bone integrity during multimodal interventions, we also assessed the reference standard, bone turnover markers (BTM) type 1 procollagen N-terminal propeptide (P1NP, Novus, Toronto, ON, Canada) and beta-C-terminal telopeptide (β-CTX, Novus, Toronto, ON, Canada) using commercial ELISA kits. P1NP is a specific indicator of type 1 collagen deposition (i.e., bone formation), while β-CTX is a sensitive marker of bone resorption (i.e., bone degradation) and may be used to detect rapid treatment responses (i.e., ~weeks to months) [43]. Thus, P1NP and β-CTX are frequently used to monitor treatment effects before bone mineral density (BMD) changes can be detected [44]. These BTM were recently chosen as primary and secondary outcome variables in a recent 1-year weight management trial on the effects of semaglutide on bone health [45].

### 2.11. Metabolic Syndrome and Sarcopenic Obesity Risk Factors

As a part of this retrospective analysis, we assessed the adaptive response to polytherapy in overweight (BMI ≥ 27) or obese (BMI ≥ 30) participants with at least one other risk factor for metabolic syndrome (obese/MetS; *n* = 20). For each participant, a ‘Global MetS Risk Index’ was quantified based on their total number of diagnostic criteria for MetS and other metabolic risk factors (min 1 and max 9), including baseline obesity (≥27 BMI, ≥28% body fat, and ≥0.9 m waist-to-hip ratio), dyslipidemia (≥1.69 mmol/L triglycerides and ≤0.9 mmol/L HDL), dysglycemia (≥5.7% HbA1c), hypertension (≥140 systolic and ≥90 diastolic), as well as systemic inflammation (≥3 mg/L CRP).

Furthermore, the diagnostic criteria for sarcopenic obesity, as established in the ESPEN and EASO consensus statement [40], were used to generate a Sarcopenic Obesity Risk Rank for all study participants. Participants were ranked according to baseline obesity (% BF; low-to-high), allometric lean mass (ASM/BW; high-to-low), 5XSTS time (low-to-high), and the Global MetS Risk Index (low-to-high), with a rank of 1 representing the lowest and 20 the highest risk for each diagnostic criteria, respectively, which were then averaged to obtain an overall Sarcopenic Obesity Risk Rank.

### 2.12. Statistical Analyses

#### 2.12.1. Retrospective Analysis 1

Multiple linear regression analyses were first run on putative predictors of the anabolic/treatment response to multimodal therapy using the full sample size of the original RCT (*n* = 32), including previously identified causes of anabolic resistance (i.e., chronological age, physical inactivity, and kidney dysfunction), metabolic risk factors (obesity, dyslipidemia, dysglycemia, hypertension, and systemic inflammation), a ‘global risk index’ for metabolic syndrome (MetS), and treatment compliance (MIS and HBRE). All independent variables, except MIS and HBRE compliances, were obtained pre-intervention.

Dependent variables were the improvements in primary and secondary outcomes, including lean mass (LM; TLM and ASM), allometric lean mass (TLM/BW, TLM/BMI, ASM/BW, and ASM/BMI), lean mass/body fat indices (TLM/FM, TLM/%BF, ASM/FM, and ASM/%BF), strength, and performance. Pre-to-post-intervention improvements were calculated as percent changes (Δ%) using the standard formula ((post-intervention test result − pre-intervention test result)/pre-intervention test result) * 100).

For all regression models, backward stepwise regression and collinearity testing were used to eliminate redundant variables and determine the best fit and strongest predictors of the adaptive response to multimodal therapy. Weakening of the overall model, negligible or weak correlation with the outcome (r < 0.2; *p* > 0.05), non-casual correlation with the outcome (nonsense), and redundancy/collinearity with other predictors were considered exclusion criteria for independent variables.

#### 2.12.2. Retrospective Analysis 2

Secondly, to assess the difference between high- vs. lower-quality MIS on the adaptive response to multimodal therapy in individuals at risk for sarcopenic obesity, we compared improvements in lean mass, strength, and performance between placebo (*n* = 8) and M5 (*n* = 12) groups in a subgroup with obesity/MetS (*n* = 20). Between-group differences in improvement (Δ%) for each outcome variable were analyzed by independent *t*-tests (* *p* ≤ 0.05; ** *p* ≤ 0.01; *** *p* ≤ 0.001; ^†^ *p* > 0.05 < 0.100). Within-group differences in pre vs. post-results for each outcome were analyzed by paired *t*-tests (^#^ *p* ≤ 0.05; ^##^ *p* ≤ 0.01; ^###^ *p* ≤ 0.001; ^‡^ *p* > 0.05 < 0.100). STATISTICA Version 8.0 (StatSoft, Tulsa, OK, USA) was used for all statistical analyses.

Group means ± SE and/or improvement (Δ%) are presented in table and figure formats, and *n*-sizes for all outcomes are shown in figure and table captions.

## 3. Results

### 3.1. Retrospective Analysis 1: Predictors of the Adaptive Response to HBRE + PA + MIS Polytherapy

**Figure 3:**
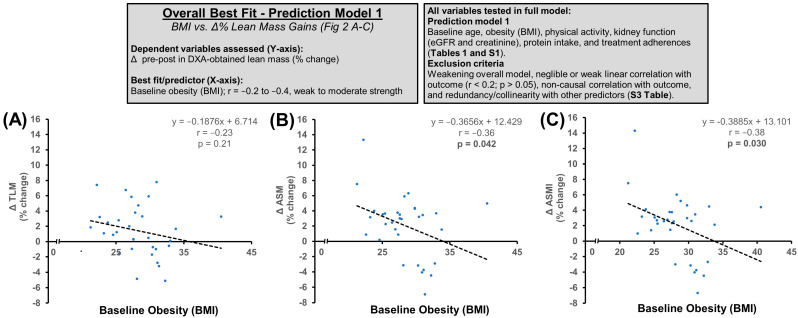
Overall best fit in prediction model 1. Baseline obesity (BMI) was the strongest individual predictor of % lean mass gains following three months of HBRE + PA + MIS polytherapy. The sample size for analyses *n* = 30–32. All correlations are shown in Table 1 and Table S1. (**A**) Baseline BMI vs. Δ TLM (% change); (**B**) Baseline BMI vs Δ ASM (% change); (**C**) Baseline BMI vs Δ ASMI (% change).

In prediction model 1, known causes of anabolic resistance (chronological age, obesity, physical inactivity, and kidney dysfunction) and other putative predictors of the adaptive response (daily protein intake and treatment compliance) were included in the full model (Table 1 and Table S1). Independent variables were then gradually eliminated by backward stepwise regression and collinearity testing (Table S3). Overall, baseline obesity provided the best fit, with pre-intervention BMI being a moderate-to-strong negative predictor of the Δ% improvements in ASM (r = −0.36, *p* = 0.043), ASMI (r = −0.38, *p* = 0.030), TLM/BW (r = −0.67, *p* < 0.001), and TLM/BMI (r = −0.56, *p* = 0.001) (Figure 3). While baseline protein intake was a moderate-to-strong positive predictor of gains in TLM/BW (r = 0.57, *p* = 0.001) and TLM/BMI (r = 0.39, *p* = 0.025), it did not improve the overall fit of the model.

**Figure 4:**
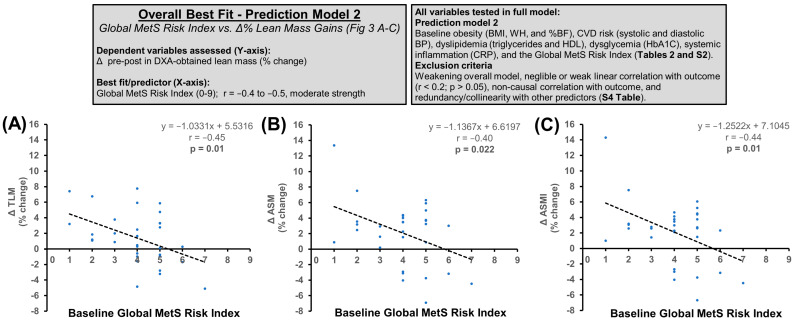
Overall best fit in prediction model 2. Compared to the individual metabolic risk factors (i.e., obesity, dyslipidemia, dysglycemia, and systemic inflammation), the Global MetS Risk Index (i.e., total MetS risk factors Section 2.11) was the overall strongest predictor of % lean mass gains following three months of HBRE + PA + MIS polytherapy. The sample size for analyses *n* = 30–32. All correlations are shown in Table 2 and Table S2. (**A**) Baseline MetS Risk Index vs. Δ TLM (% change); (**B**) Baseline MetS Risk Index vs. Δ ASM (% change); (**C**) Baseline MetS Risk Index vs. Δ ASMI (% change).

In the next model, diagnostic criteria for metabolic syndrome (MetS; obesity, dyslipidemia, dysglycemia, and hypertension), other metabolic risk factors (systemic inflammation), and a ‘global risk index’ for MetS (i.e., total risk factors) were included in the full model (Table 2 and Table S2). Redundant variables were removed by collinearity testing (Table S4), and the best fit was determined by backward stepwise regression. The Global MetS Risk Index provided the best overall fit being a moderate-to-strong negative predictor of the Δ% gains in lean mass (TLM, r = −0.45, *p* = 0.010; ASM, r = −0.40, *p* = 0.023), allometric lean mass (ASMI, r = −0.45, *p* = 0.011; TLM/BW, r = −0.53, *p* = 0.002; TLM/BMI, r = −0.37, *p* = 0.037) and lean mass/body fat indices (TLM/%BF, r = −0.40, *p* = 0.022; ASM/%BF, r = −0.42, *p* = 0.016) (Figure 4).

Among individual MetS diagnostic criteria, baseline obesity (BMI, %BF, and WH) and dyslipidemia (triglycerides and HDL) were moderate-to-very strong, negative predictors of Δ% gains in ASM, ASMI, TLM/BW and TLM/BMI (*p* < 0.05). Furthermore, HDL was positively correlated with % gains in lean mass, allometric lean mass, and lean mass/body fat indices, while other diagnostic criteria for MetS were negatively correlated with the adaptive response. 

### 3.2. Retrospective Analysis 2: Adaptive Response to HBRE + PA and High-Quality or Lower-Quality MIS in Free-Living Older Adults with Obesity/MetS

Next, we performed a subgroup analysis of participants who were overweight (BMI ≥ 27) or obese (BMI ≥ 30) with at least one other MetS risk factor (obese/MetS, *n* = 20). Our aim was to compare the adaptive response to polytherapy with either higher- (M5; whey + casein, creatine monohydrate, vitamin D_3_, calcium and fish oil, *n* = 12) or lower-quality (PLA; collagen + safflower oil, *n* = 8), protein-based MIS, with both groups either matching or exceeding current expert recommendations on total, daily protein intake (1.2–1.5 g/kg BW/d) [4,46].

### 3.3. Anthropometry and Vital Signs

At baseline, the obese/MetS subgroup exhibited several diagnostic criteria for MetS (“Global MetS Risk Index”) and were polymorbid and at risk for sarcopenic obesity. Thus, both PLA and M5 groups were representative of free-living older males in North America [25,26] and well-matched in terms of age, anthropometry and overall health status (Table 3).

Following the intervention, body weights and BMIs were moderately increased in both groups, with pre-to-post results borderline significant in the PLA group (*p* > 0.05 < 0.100). Vital signs were largely unaffected by HBRE + PA + MIS polytherapy, while diastolic BP significantly increased in the M5 group pre-to-post intervention (*p* < 0.05).

### 3.4. Compliance, PA Goals, and Dietary Intake

Compliance with the HBRE intervention was comparable to previously published unsupervised RCTs [29] and not significantly different between PLA and M5 groups (89% vs. 76%, respectively) (Table 4). Collectively, participants followed the ACSM guidelines for full-body strength training for older adults and increased elastic band resistance progressively over the 3-month intervention (*p* < 0.05).

The average daily step goal was chosen for improving health-related QoL and obesity/MetS status [34] but was not met by either cohort. While the M5 group marginally improved step counts over the duration of the study (the equivalent of ~400 m/day), the PLA group significantly decreased step counts pre-to-post intervention (the equivalent of ~1000 m/day) (*p* < 0.05). These PA levels were technically within the normal range for this population (2000–9000 steps), but both groups qualified as low-active/sedentary throughout the study [34].

Supplement adherences corresponded well to previously published RCTs [47] and were not significantly different between PLA and M5 groups (95% vs. 89%, respectively) (Table 5).

Macronutrient analyses of 72-h dietary recalls (food only) indicated that energy and carbohydrate intakes were significantly higher in the M5 as compared to the PLA group at baseline (*p* < 0.05) (Table 6), while there were no differences in protein or fat intakes. Participants were encouraged to maintain their dietary habits, but food consumption was significantly increased pre-to-post intervention in the PLA group, resulting in an overall higher energy intake (*p* < 0.05) (net positive energy balance). In contrast, food consumption was modestly decreased in the M5 group, decreasing overall energy intake (*p* < 0.05).

As designed, total protein consumption (food + supplement) was significantly increased in both groups pre-to-post intervention (PLA, 1.43 ± 0.09 g/kg BW; M5, 1.26 ± 0.09 g/kg BW) and exceeded the current RDA (0.8 g/kg BW/d) and expert recommendations on optimal protein intake for older adults (≥1.2 g/kg BW/d) [46,48,49,50], thus, meeting the proposed guidelines for sarcopenic obesity (1.2–1.5 g/kg/BW/day) [4] and weight management (1.2–1.6 g/kg BW/d) [46,51]. Although total protein intake was higher in PLA vs. M5, it was not statistically different between groups (*p* = 0.22).

### 3.5. Blood Chemistry

Markers of metabolic disease (i.e., dyslipidemia, dysglycemia and systemic inflammation) were measured pre and post intervention, including bloods lipids, hemoglobin A1c, and C-reactive protein. While not statistically significant, these risk markers were consistently reduced and increased, respectively, in the M5 vs. PLA group (Table 7). These diametrically opposing effects were confirmed directionally by the Global Risk Index for MetS (i.e., total MetS risk factors; Table 3) which was determined to be a key driver of anabolic resistance at old age (Section 3.1 and Section 3.2).

Furthermore, P1NP levels and the P1NP/CTX ratio were increased in the M5 group only, indicative of improved bone turnover and bone formation following three months of HBRE + PA with high-quality, protein-based MIS. While a longer duration trial is necessary to confirm BMD changes (i.e., 6 months–2 years), P1NP is a specific marker of type 1 collagen deposition and a sensitive indicator of bone formation that is used for tracking treatment efficacy in short-duration, interventional RCTs (weeks-months) [43].

Clinical markers of liver (bilirubin, ALT, and GGT) and kidney (creatinine and eGFR) function indicated no toxic effects of MIS in either group (Table S6). Notably, an increase in creatinine excretion is to be expected following significant LBM gain and creatine supplementation, such as in the M5 group. Creatinine is a natural byproduct of creatine breakdown and in line with muscle levels of creatine and phosphocreatine [52]. Because eGFR is calculated using serum creatinine levels, a reduction in eGFR is not an indication of decreased kidney function in this case, but rather creatine loading and muscle anabolism.

### 3.6. Δ Lean Mass, Lean Mass/Body Fat Indices, and Allometric Lean Mass

**Figure 5:**
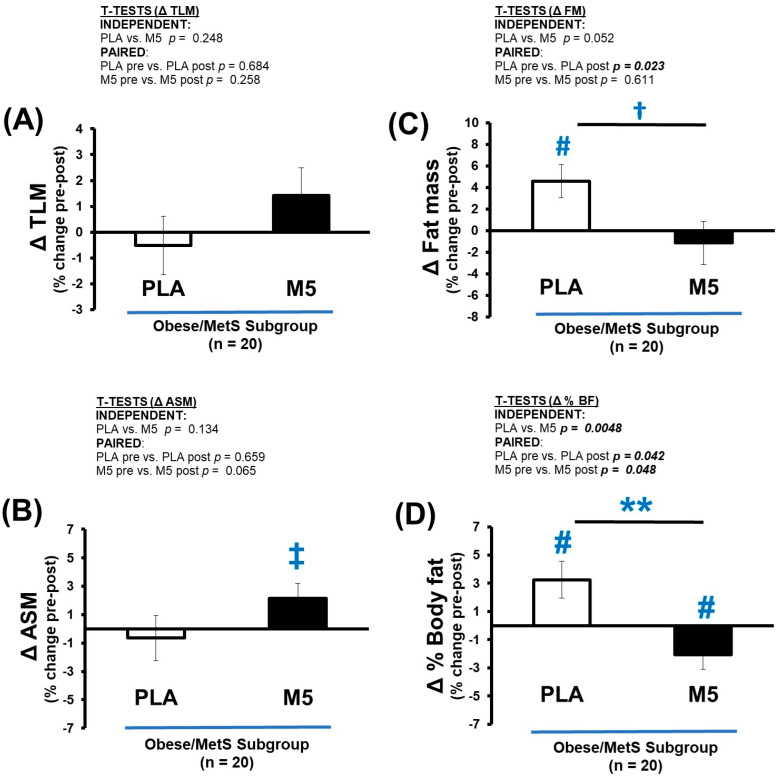
Δ LM, FM, and % BF. Between-group differences in the adaptive response (i.e., Δ% changes) were analyzed by independent *t*-tests (** *p* ≤ 0.01; ^†^ *p* > 0.05 < 0.100). Within-group differences in pre-post intervention results were analyzed by paired *t*-tests (^#^ *p* ≤ 0.05; ^‡^ *p* > 0.05 < 0.100) (Table 8). Sample size for analyses *n* = 20 (PLA; *n* = 8, M5; *n* = 12). (**A**) Δ TLM (% change); (**B**) Δ ASM (% change); (**C**) Δ FM (% change); (**D**) Δ% Body fat (% change).

**Figure 6:**
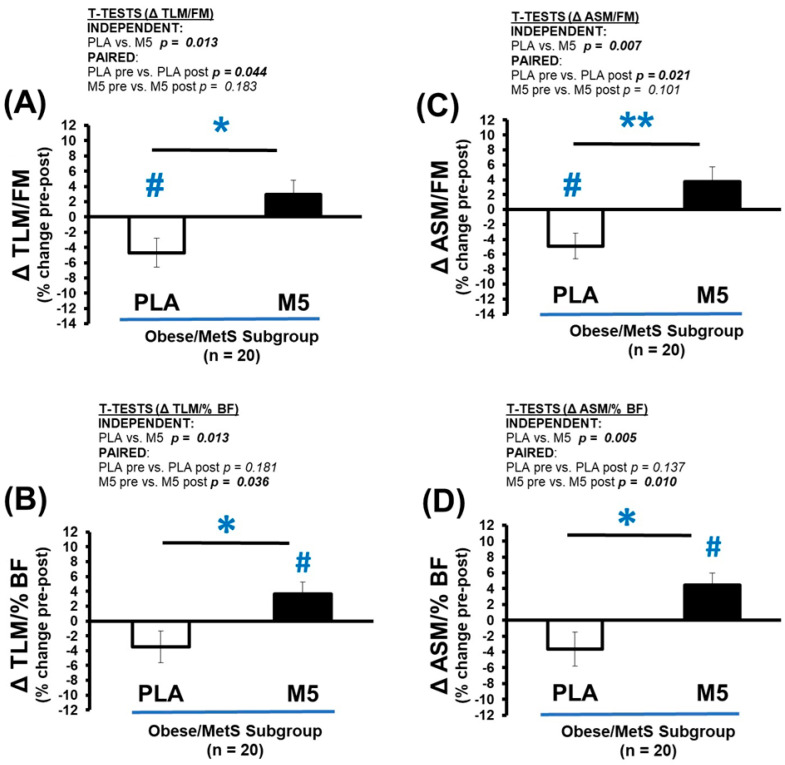
Δ Body Composition Indices. Between-group differences in the adaptive response (i.e., Δ% changes) were analyzed by independent *t*-tests (* *p* ≤ 0.05; ** *p* ≤ 0.01). Within-group differences in pre-post intervention results were analyzed by paired *t*-tests (^#^ *p* ≤ 0.05) (Table 8). Sample size for analyses *n* = 20 (PLA; *n* = 8, M5; *n* = 12). (**A**) Δ TLM/FM (% change); (**B**) Δ TLM/%BF (% change); (**C**) Δ ASM/FM (% change); (**D**) Δ ASM/%BF (% change).

The M5 group showed moderate Δ gains in TLM and ASM, a marginal loss of fat mass, and significantly improved % BF (*p* < 0.05) (Table 8 and Figure 5A–D). Concurrently, the PLA group exhibited marginal losses of TLM and ASM, combined with significant gains in FM (*p* < 0.05) and %BF (*p* < 0.05). Predictably, these opposing outcomes between-groups yielded significantly different Δ changes in lean mass/body fat ratios, including TLM/FM (*p* < 0.05), TLM/%BF (*p* < 0.05), ASM/FM (*p* < 0.05), and ASM/%BF (*p* < 0.05) (Figure 6A–D).

The appendicular skeletal muscle mass index (ASMI; ASM/h^2^) is a key diagnostic measure for sarcopenia diagnosis, associated with lower mortality risk in obese individuals, and positively correlated to leukocyte telomere length [38,39,53,54]. Because of a modest gain in appendicular lean mass, ASMI was improved in the M5 group (*p* > 0.05 < 0.100), while it was marginally reduced in the PLA group following the intervention.

**Figure 7:**
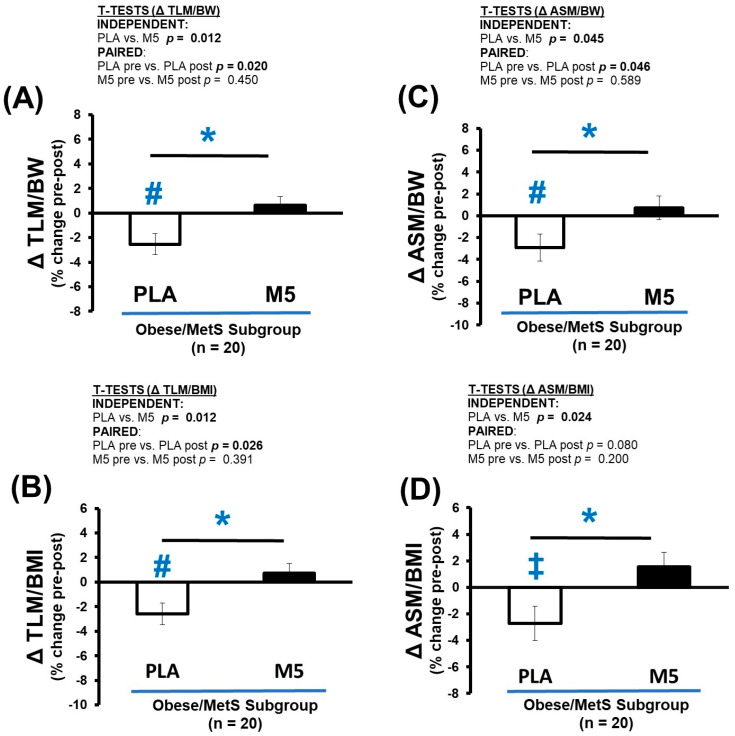
Δ Allometric LM. Between-group differences in the adaptive response (i.e., Δ% changes) were analyzed by independent *t*-tests (* *p* ≤ 0.05). Within-group differences in pre-post intervention results were analyzed by paired *t*-tests (^#^ *p* ≤ 0.05; ^‡^ *p* > 0.05 < 0.100) (Table 8). Sample size for analyses *n* = 20 (PLA; *n* = 8, M5; *n* = 12). (**A**) Δ TLM/BW (% change); (**B**) Δ TLM/BMI (% change); (**C**) Δ ASM/BW (% change); (**D**) Δ ASM/BMI (% change).

The DXA outcomes of most clinical relevance for sarcopenic obesity, i.e., allometric lean mass, significantly worsened pre-to-post intervention in the PLA group, including TLM/BW (*p* < 0.05), TLM/BMI (*p* < 0.05), ASM/BW (*p* < 0.05), and ASM/BMI (*p* > 0.05 < 0.10) (Figure 7A–D). Meanwhile, allometric lean mass was moderately improved in the M5 group; thus, the Δ% changes were significantly different between-groups (*p* < 0.05).

### 3.7. Δ Strength

**Figure 8:**
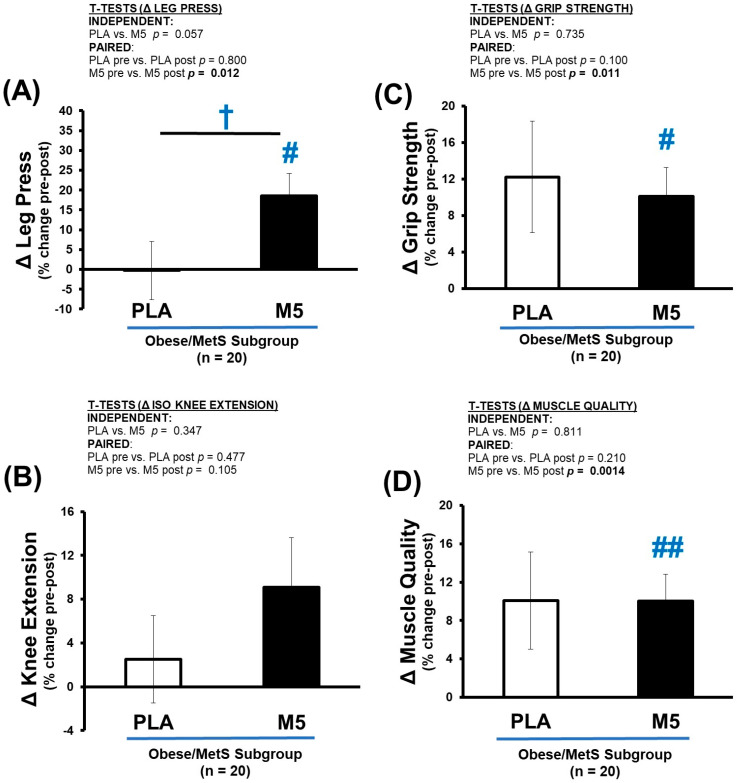
Δ Strength and Muscle Quality. Between-group differences in the adaptive response (i.e., Δ% changes) were analyzed by independent *t*-tests (^†^ *p* > 0.05 < 0.100). Within-group differences in pre-post intervention results were analyzed by paired *t*-tests (^#^ *p* ≤ 0.05; ^##^ *p* ≤ 0.01) (Table 9). Sample size for strength outcomes *n* = 19 (PLA; *n* = 7, M5; *n* = 12). (**A**) Δ Leg Press (% change); (**B**) Knee Extension (% change); (**C**) Grip Strength (% change); (**D**) Δ Muscle Quality (% change).

Both maximal grip and leg press results were significantly improved in the M5 group pre-to-post intervention (*p* < 0.05), combined with a moderate improvement in isometric knee extension (*p* = 0.105) (Table 9 and Figure 8A–C). As expected, the PLA group also exhibited some improvement in strength (grip) following three months of HBRE, but this did not reach statistical significance because of interindividual variance within the group. Collectively, the strength improvements were more robust and uniform in the M5 vs. PLA group, which was also indicated in the muscle quality (MQ) index (Figure 8D).

### 3.8. Δ Performance

**Figure 9:**
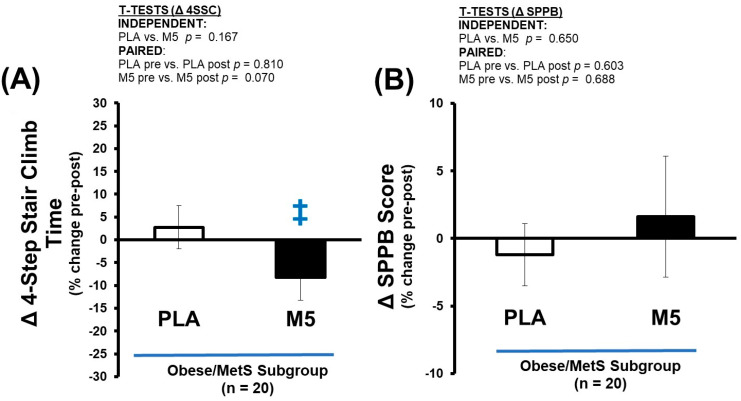
Δ Performance. Between-group differences in the adaptive response (i.e., Δ% changes) were analyzed by independent *t*-tests (no significance detected). Within-group differences in pre-post intervention results were analyzed by paired *t*-tests (^‡^ *p* > 0.05 < 0.100) (Table 10). Sample size for performance outcomes *n* = 19 (PLA; *n* = 7, M5; *n* = 12). (**A**) Δ 4SSC (% change); (**B**) Δ SPPB Score (% change).

Albeit not reaching statistical significance *per se*, the time to complete the functional mobility tests was modestly improved pre-to-post intervention in the M5 group (4SSC; *p* = 0.24 and 5XSTS; *p* > 0.05 < 0.10), but not in the PLA group (Table 10 and Figure 9). Other components of the SPPB, such as balance testing and walk speed, were largely unchanged in both groups.

### 3.9. Ranks of Anabolic Response

The adaptive response (Δ%) to the multimodal intervention for each participant was ranked from 1 (lowest responder) to 20 (highest responder) for lean mass (LM, allometric LM, and LM/BF ratios), strength (isometric knee extension and leg press) and performance (4SSC and SPPB score). An overall rank of the anabolic response could then be generated from the average ranks of Δ lean mass, Δ strength, and Δ performance.

**Figure 10:**
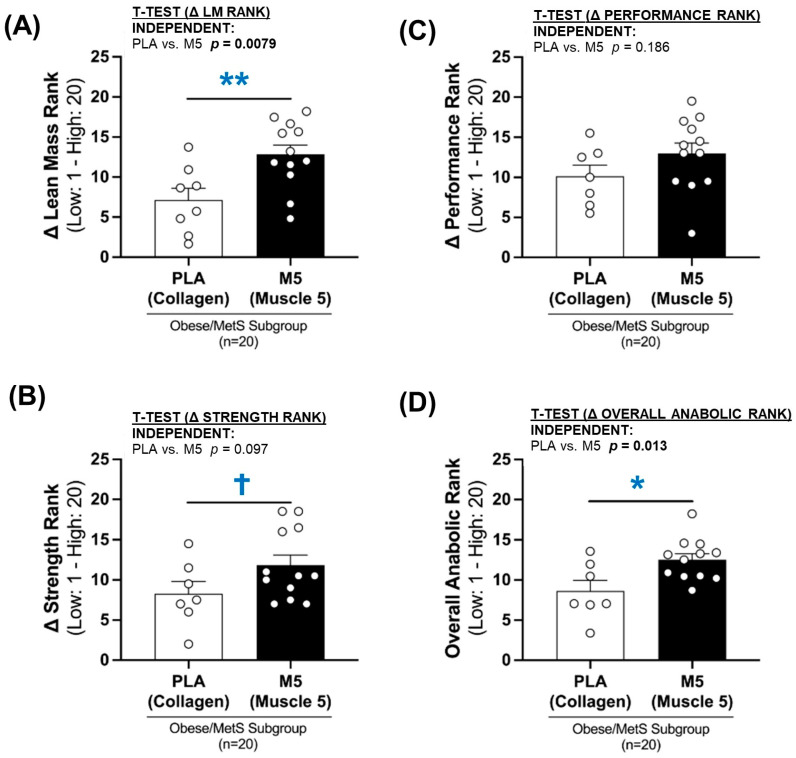
Anabolic Ranks. Ranks (low 1- high 20) of anabolic responses (Δ lean mass, Δ strength, Δ performance, and Δ overall) in high-quality (*n* = 12; M5 + fish oil) vs. lower-quality (*n* = 8; PLA; collagen + safflower oil) supplement groups in obese/MetS older males following three months of home-based resistance exercise and daily walking (HBRE + PA). Between-group differences in the anabolic response ranks were analyzed by independent *t*-tests (* *p* ≤ 0.05; **p ≤ 0.01; ^†^ *p* > 0.05 < 0.100). Sample size for anabolic ranks *n* = 19–20 (PLA; *n* = 7–8, M5; *n* = 12). The rankings for lean mass, strength, performance and overall anabolic response are defined in Section 3.9. (**A**) Δ Lean Mass Rank (1–20); (**B**) Δ Strength Rank (1–20); (**C**) Δ Performance Rank (1–20); (**D**) Overall Anabolic Rank (1–20).

For each area of assessment, the M5 group consistently ranked higher than the PLA group (rank Δ lean mass, *p* = 0.0079; rank Δ strength, *p* = 0.097; rank Δ performance, *p* = 0.186) (Figure 10A–C), suggestive of a more robust overall anabolic response in the high-quality protein group (*p* < 0.013) (Figure 10D).

### 3.10. Sarcopenic Obesity Risk

**Figure 11:**
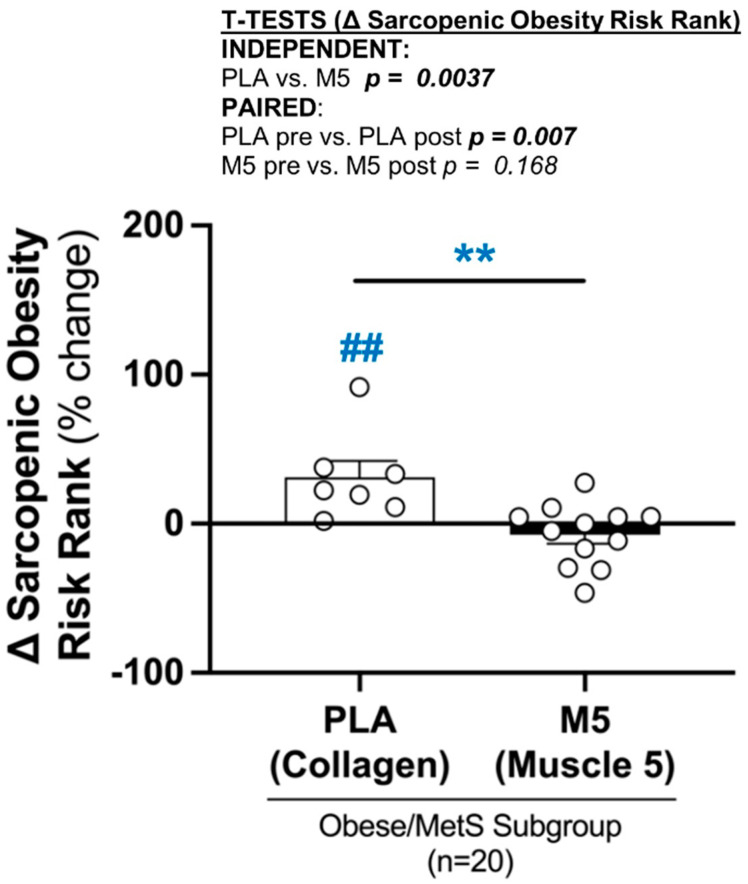
Δ Sarcopenic Obesity Risk Rank. Between-group differences in the adaptive response (i.e., Δ% changes) were analyzed by independent *t*-tests (** *p* ≤ 0.01). Within-group differences in pre-post intervention results were analyzed by paired *t*-tests (^##^ *p* ≤ 0.01) (Table 3). The Sarcopenic Obesity Risk Rank is defined in Section 2.11. Sample size for anabolic ranks *n* = 19 (PLA; *n* = 7, M5; *n* = 12).

The differential adaptive response to HBRE + PA + MIS polytherapy between high- (M5 + fish oil) vs. lower-quality (collagen + safflower oil) supplement groups was also reflected in the Sarcopenic Obesity Risk Rank, with a significant increase vs. modest decrease in PLA (*p* < 0.01) vs. M5 (*p* > 0.05), respectively (Δ% between-groups *p* < 0.01) (Figure 11 and Table 3).

**Figure 12:**
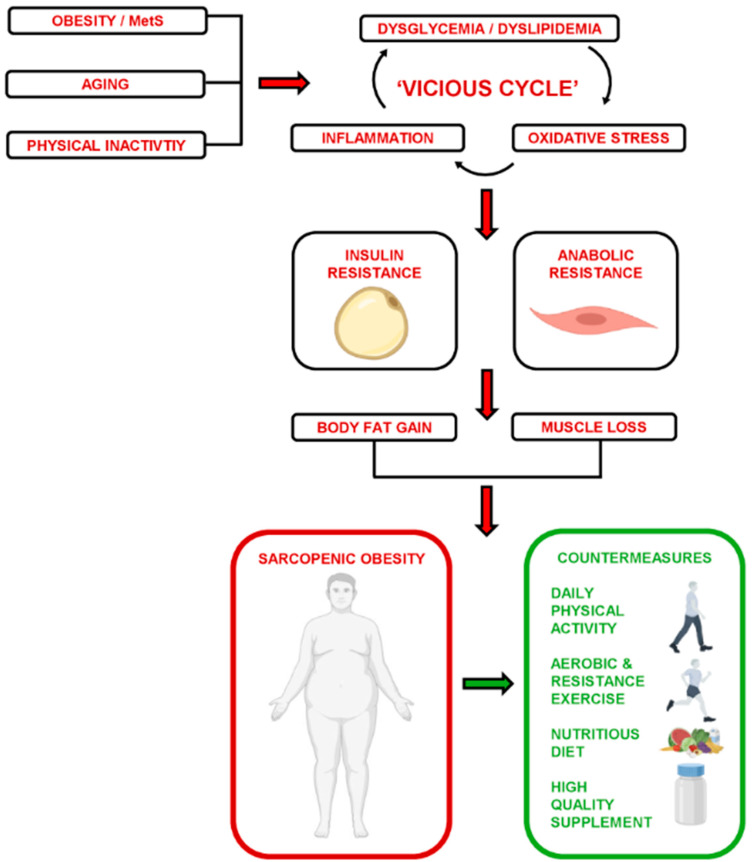
Drivers and countermeasures of anabolic resistance in sarcopenic obesity.

Taken all together, these results suggest that the combined benefits of resistance exercise, higher-quality MIS (i.e., whey/casein > collagen peptides), and lifestyle modification (PA and diet) are necessary to mitigate muscle deterioration and sarcopenic obesity risk in older, polymorbid individuals (Figure 12).

## 4. Discussion

In this retrospective analysis, we demonstrated that the long-term adaptive response to home-based resistance exercise (HBRE), daily physical activity (PA), and multi-ingredient supplementation (MIS) was negatively correlated with pre-intervention obesity and total MetS risk factors (Global MetS Risk Index) in a free-living cohort of older men. Specifically, obesity and the Global MetS Risk Index were moderate-to-strong, negative predictors of the adaptive response to polytherapy, while baseline chronological age, kidney function, and physical activity levels were only weakly associated with outcomes, suggesting that obesity and metabolic disease are key drivers of anabolic resistance at old age. Furthermore, we showed that a higher-quality, protein-based MIS (M5; whey/casein, creatine, calcium, vitamin D_3_, and fish oil) provided superior lean mass, strength and performance adaptations as compared to a lower-quality alternative (PLA; collagen peptides and safflower oil) in obese/MetS individuals undergoing HBRE + PA + MIS polytherapy.

The main findings from the regression analyses are largely consistent with those from tracer studies assessing the acute anabolic response to protein feeding and/or contractile activity in obese humans and animal models [55,56,57,58,59]. Work by Nilsson and Fluckey demonstrated a blunted 24-h protein synthetic response (MPS) to resistance exercise in various subfractions of fast-twitch muscles from sarcopenic obese rats [58]. Similar results have been reported in both young and old obese humans, including the recent works of Murton and Greenhaff [59], Beals and Burd [55,56], and Smeuninx and Breen [57]. While chronological age, PA levels, and kidney function were only weakly correlated to the long-term adaptations to HBRE + PA + MIS polytherapy, there are plausible explanations for this. Compared to end-stage renal disease (eGFR < 15 mL/min) (reviewed in [1]), age-associated kidney impairment is likely too mild to affect the long-term adaptive response. Secondly, we are not aware of any studies demonstrating that anabolic resistance becomes progressively worse across older age categories, such as septuagenarians (70–79 years old), octogenarians (80–89 years old), and nonagenarians (90–99 years old). Our cohort ranged from 66 to 91 years of age, and our findings do not support the notion that chronological age is a significant driver of anabolic resistance across these age categories. Third, it is well known that PA protects against age-related muscle loss, and Smeuninx and Breen recently reported a positive correlation between daily step counts and the MPS response to protein feeding in older adults [57]. Surprisingly, we did not find that baseline PA was significantly associated with the adaptive response, although our trial was designed to assess long-term adaptations rather than acute signaling events or MPS. Taken all together, we surmise that obesity and associated metabolic risk factors are the strongest contributors to SM anabolic resistance in old age and impair both the acute and long-term response to exercise- and supplement-based therapies.

Next, we demonstrated that the adaptive response to HBRE + PA + MIS was dependent on the quality of the multi-ingredient supplement in a subgroup of overweight/obese participants at risk for sarcopenic obesity (obese/MetS). Consistently, the higher-quality, protein-based MIS group (M5) exhibited greater Δ% gains in all clinically relevant outcomes, including body composition, strength, and performance vs. the PLA group (i.e., lower-quality MIS), although both cohorts exceeded current recommendations for daily protein intake in older adults. Furthermore, obesity-associated metabolic risk factors were modestly improved in the M5 group only, resulting in a significant decrease in the Sarcopenic Obesity Risk Rank vs. the PLA group. While the PLA-treated participants also improved strength and muscle quality, their overall adaptive response was significantly attenuated despite being compliant with HBRE + MIS and with an overall daily protein consumption that exceeded the M5 group (PLA, 1.43 ± 0.09 g/kg BW; M5, 1.26 ± 0.09 g/kg BW), and in line with expert recommendations on optimal protein intake for sarcopenic obesity (1.2–1.5 g/kg/BW/day). These results are consistent with those studies showing a superior MPS response [36,37], greater LBM regain during recovery from energy restriction and physical inactivity [60], and more robust adaptations to exercise interventions in whey- (or whey/casein) vs. collagen-supplemented groups [22,47]. Notably, hydrolyzed collagen has been used as an isocaloric, protein-matched placebo by our research team because of its inferior anabolic potential, including low levels of methionine (i.e., rate limiting for translation initiation), EAAs, BCAAs, and poor amino acid digestibility score (DIAAS of 0). Thus, our results are in contrast to those of Jendricke, Zdzieblik and König, who have reported generally superior results for collagen peptide vs. whey protein supplementation upon body composition, bone health, and knee joint discomfort in a series of studies [61,62,63,64,65]. Currently, we, and others [66], are unable to explain this discrepancy, and we refer the readers to the excellent reviews performed by Deane and Atherton and Holwerda and van Loon for more information on collagen and its therapeutic potential on muscle, bone, and connective tissue [35,67].

A limitation of the current study with respect to exercise-induced energy expenditure is that the daily step goals were not met by the participants and that the collagen-supplemented group increased their daily energy intake during the intervention (i.e., net positive energy balance). While speculative, an increased food intake may be a compensatory response to engaging in a chronic exercise program in low-active/sedentary individuals. Specifically, various forms of strength training have been shown to increase the drive to eat and alter appetite-regulatory hormones in obese males [68]. Conversely, daily energy intake was modestly reduced in the M5 group, potentially indicative of a greater satiety-inducing effect from daily consumption of humanized milk proteins (whey/casein; 60:40 ratio) vs. collagen peptides. In support of our data, Zdzieblik and König’s RCT also showed that collagen peptides increased daily energy intake in middle-aged, untrained males undergoing 12 weeks of resistance training [63], yet it was decreased in the control and whey-supplemented groups. Currently, it is well-accepted that proteins are beneficial for regulating appetite and energy expenditure, but it is unclear which protein source is more satiating in the long term [69]. Interestingly, reversing the whey/casein ratio in cowmilk (20:80) to resemble human breast milk (70:30) has been shown to augment plasma AUCs of insulin, C-peptide, incretins (GLP-1 and GIP), total amino acids (AAs), and branched-chain AAs following ingestion [70]. Evidence also points to the co-ingestion of protein or specific AAs with calcium, providing a potent synergy on GLP-1 release and appetite suppression [71]. Thus, these observations may suggest that the whey/casein ratio in the M5 supplement (“humanized milk ratio”), combined with the calcium caseinate, is optimized for inducing satiety and anabolism compared to an incomplete protein source. Notwithstanding, we surmise that the home-based intervention affected overall compliance negatively (specifically, daily step goals and food intake) and that involuntary compensatory mechanisms, such as satiety-signaling, may have contributed to the observed outcomes in the obese/MetS cohort.

While not designed as a weight loss trial per se, the current study may also be of interest for LBM maintenance in weight management, with specific relevance to individuals at risk for sarcopenic obesity. All types of weight management, such as bariatric surgery, pharmacotherapy, and low-calorie diets, lead to substantial FFM loss [10,12,16,17,72], typically exceeding the widely cited ‘Quarter FFM Rule’ [73] and accounting for ≥20–40% of total weight loss [17]. With the meteoric rise in the use of GLP-1RA and GLP-1RA/GIP dual agonists, there has been a renewed interest in developing countermeasures for maintaining LBM in the face of rapid weight loss. For example, it has been demonstrated that even combining aerobic and resistance exercise (or each on their own), protein intake of 1.0 g/kg BW/d, and daily supplementation with vitamin D (1000 IU/d) and calcium (1500 mg/d) is not sufficient to prevent LBM loss during caloric restriction (−500 to −750 kcal/d) in obese, older adults [74,75]. Considering that weight loss is more rapid and extreme in bariatric surgery and pharmacotherapy, it cannot be expected that just an ‘adequate’ daily protein intake (i.e., 0.8–1 g/kg BW/d) without provision of extra high-quality proteins (e.g., whey and casein) to match or exceed recommended daily protein intake (1.2–1.6 g/kg BW/d), would prevent muscle deterioration with GLP-1RA and GLP-1RA/GIP dual agonist use. Our study also suggests that increasing the protein intake from mixed sources (0.84 g/kg/d) by over 50% (to 1.43 g/kg/d) using a lower quality protein source (collagen peptides) does not yield positive effects upon satiety/food intake vs. a high-quality protein source. Thus, when applying multimodal interventions for weight management, it is important to consider both protein quality (whey/casein > collagen > plant) and quantity (1.2–1.6 g/kg BW/d).

Concurrent with LBM loss, bone deterioration is a significant issue in weight management, which is particularly relevant for older adults who have difficulty regaining bone mass and are at higher risk for fractures, falls, and serious injuries [76,77]. Large-scale epidemiological studies have consistently shown that weight loss over 6–12 months is associated with a ~2% reduction in bone mineral density (BMD) and higher fracture risk in older adults, including overweight and obese individuals [77]. Studies also indicate that potential countermeasures, such as weight-bearing exercise and resistance training, do not fully protect against LBM and bone deterioration, as exemplified by the Villareal studies [74,75]. Furthermore, when bone mass is lost, it does not seem to be regained in full even after weight regain [77]. In terms of robust weight loss solutions, GLP1-RA therapy (i.e., semaglutide and liraglutide) and bariatric surgery significantly reduce spine and hip BMDs [15,45,77,78]. Specifically, bariatric surgery is associated with substantial biochemical, hormonal, and mechanical changes and reduces BMD by ~5–10% at the spine and hip, thus increasing fracture risk [78,79,80]. While the mechanisms of bone loss are multiple, bone resorption, as measured by CTX levels, is increased 6 months onward during pharmacotherapy (semaglutide [45]) and persists even 2–3 years following bariatric surgery [78], further stressing the importance of developing new countermeasures for LBM and bone loss during weight management. AACE/TOS/ASMBS clinical practice guidelines currently stipulate supplementation with calcium citrate and vitamin D_3_ to attenuate bone loss following surgical weight loss [78]. Muschitz et al. found that calcium and vitamin D_3_ significantly attenuated bone loss following bariatric surgery when combined with protein supplementation and exercise [81]. Our study also suggests that a whey/casein-based MIS that contains creatine, vitamin D_3_, calcium and fish oil may be ideal for maintaining bone health in overweight and obese individuals who undergo exercise-based therapy, although a longer duration, dedicated weight-loss trial is necessary to confirm structural bone benefits.

While there are many benefits to using an unsupervised study design and multi- vs. single-ingredient supplementation, we certainly acknowledge that the current trial has several limitations. First, it is not possible to determine the individual contributions of each component of M5 to the observed outcomes; however, the ingredients were carefully chosen for their independent benefits on LBM, body composition (muscle-to-fat ratios), bone accretion, and/or metabolic risk factors. We have now also shown that this combination of ingredients improves LBM, strength/function, bone formation, and metabolic risk factors under both controlled and free-living conditions [22,47]. Secondly, the sample size for the current analysis was modest (*n* = 20), increasing overall variance and the likelihood of type 2 error. However, the results consistently favored M5 over the PLA group, which was clearly indicated in the individual and overall anabolic response ranks (Figure 10). While the observed outcomes may have been partially driven by involuntary compensatory mechanisms affecting daily PA and food intake levels, this is an inherent risk of unsupervised trials. We also surmise that the long-term, satiety-inducing effect of consuming whey/casein over collagen is a true phenomenon grounded in evolutionary biology that deserves to be studied in more detail, especially in the context of body re-composition and weight management. For millions of years, mammalian species have consumed milk for satiety, growth and development [82], and modern humans have also consumed milk from other species, such as cows, buffaloes, goats, and camels, since the Neolithic revolution (~10,000 years ago) [83]. Thus, casein and whey are outstanding functional components of milk that have co-evolved to optimize satiety and growth. Lastly, there is a definite need for more RCTs on the role of protein quality (milk vs. plant vs. collagen vs. protein blends) on musculoskeletal health, training adaptations, and recovery, particularly in females. Future research should aim to optimize exercise and nutritional interventions, such as whey/casein-based MIS, for improving body composition, health, and overall well-being in this population, specifically.

## 5. Conclusions

In conclusion, our study confirms that obesity and associated metabolic risk factors are the main drivers of anabolic resistance in old age and impair the long-term adaptive response to exercise- and supplement-based interventions. We also demonstrate that a higher-quality, protein-based multi-ingredient supplement (Muscle 5; whey/casein + creatine + calcium + vitamin D_3_ + fish oil) confers superior lean mass, strength, and performance adaptations vs. a lower-quality alternative (collagen peptides + safflower oil) and attenuates sarcopenic obesity risk in polymorbid, older adults. Based on these and other findings, management strategies for mitigating concurrent LBM loss and body fat gain in aging, obesity, and polymorbid disease states (e.g., sarcopenic obesity) should ideally incorporate a higher-quality, protein-based MIS for optimizing body composition and overall health.

## Figures and Tables

**Table 1:** Full prediction model 1: Baseline predictors and correlations to the adaptive response to HBRE + PA + MIS polytherapy. Correlation coefficients (r) in bold are statistically significant (*p* ≤ 0.05).

		PREDICTION MODEL 1										
		Δ LEAN MASS (% Pre-Post)		Δ ALLOMETRIC LEAN MASS (% Pre-Post)					Δ LEAN MASS/BODY FAT INDICES (% Pre-Post)			
BASELINE PREDICTORS		∆ TLM	∆ ASM	∆ ASMI	∆ TLM/BW	∆ TLM/BMI	∆ ASM/BW	∆ ASM/BMI	∆ TLM/FM	∆ TLM/% FM	∆ ASM/FM	∆ ASM/% FM
**1. Age (years)**		r = 0.06	r = 0.18	r = 0.20	r = 0.32	r = 0.13	r = 0.18	r = 0.21	r = 0.13	r = 0.15	r = 0.20	r = 0.23
		*p* = 0.745	*p* = 0.330	*p* = 0.270	*p* = 0.071	*p* = 0.467	*p* = 0.317	*p* = 0.238	*p* = 0.480	*p* = 0.419	*p* = 0.275	*p* = 0.208
**2. Obesity (BMI)**		r = −0.23	**r = −0.36**	**r = −0.38**	**r = −0.67**	**r = −0.56**	r = −0.33	r = −0.29	r = −0.06	r = −0.11	r = −0.15	r = −0.22
		*p* = 0.212	***p* = 0.043**	***p* = 0.030**	***p* = 0.000**	***p* = 0.001**	*p* = 0.067	*p* = 0.106	*p* = 0.747	*p* = 0.536	*p* = 0.407	*p* = 0.234
**3. PA (steps/day)**		r = 0.03	r = −0.14	r = −0.10	r = 0.15	r = 0.22	r = −0.12	r = −0.21	r = 0.03	r = 0.06	r = −0.05	r = −0.03
		*p* = 0.893	*p* = 0.450	*p* = 0.597	*p* = 0.442	*p* = 0.204	*p* = 0.530	*p* = 0.258	*p* = 0.894	*p* = 0.754	*p* = 0.781	*p* = 0.892
**4. Kidney Function**												
	**A. eGFR (mL/min/1.73 m^2^)**	r = 0.16	r = 0.11	r = 0.12	r = −0.05	r = 0.04	r = 0.01	r = −0.07	r = −0.23	r = −0.08	r = −0.24	r = −0.09
		*p* = 0.399	*p* = 0.567	*p* = 0.535	*p* = 0.794	*p* = 0.810	*p* = 0.961	*p* = 0.704	*p* = 0.204	*p* = 0.684	*p* = 0.193	*p* = 0.649
	**B. Creatinine (μmol/L)**	r = −0.19	r = −0.13	r = −0.15	r = −0.01	r = −0.07	r = −0.02	r = 0.06	r = 0.20	r = 0.03	r = 0.20	r = 0.04
		*p* = 0.316	*p* = 0.476	*p* = 0.413	*p* = 0.975	*p* = 0.705	*p* = 0.915	*p* = 0.765	*p* = 0.290	*p* = 0.882	*p* = 0.277	*p* = 0.848
**6. Protein Intake (g/kgBW/d)**		r = −0.05	r = 0.10	r = 0.10	**r = 0.57**	**r = 0.39**	r = 0.15	r = 0.19	r = −0.02	r = −0.04	r = 0.05	r = 0.04
		*p* = 0.765	*p* = 0.597	*p* = 0.581	***p* = 0.001**	***p* = 0.025**	*p* = 0.403	*p* = 0.295	*p* = 0.897	*p* = 0.835	*p* = 0.770	*p* = 0.793
**7. Exercise Compliance (%)**		r = 0.20	r = 0.23	r = 0.24	r = 0.19	r = 0.08	r = 0.20	r = 0.12	r = 0.09	r = 0.18	r = 0.12	r = 0.21
		*p* = 0.291	*p* = 0.229	*p* = 0.201	*p* = 0.322	*p* = 0.659	*p* = 0.282	*p* = 0.511	*p* = 0.618	*p* = 0.354	*p* = 0.513	*p* = 0.263
**8. Supplement Compliance (%)**		r = −0.02	r = −0.01	r = 0.03	r = 0.02	r = 0.04	r = −0.18	r = −0.17	r = −0.14	r = −0.11	r = −0.14	r = −0.11
		*p* = 0.931	*p* = 0.979	*p* = 0.866	*p* = 0.922	*p* = 0.833	*p* = 0.349	*p* = 0.382	*p* = 0.465	*p* = 0.572	*p* = 0.460	*p* = 0.573

**Table 2:** Full prediction model 2: Baseline predictors and correlations to the adaptive response to HBRE + PA + MIS polytherapy. Correlation coefficients (r) in bold are statistically significant (*p* ≤ 0.05).

		PREDICTON MODEL 2										
		Δ LEAN MASS (% Pre-Post)		Δ ALLOMETRIC LEAN MASS (% Pre-Post)					Δ LEAN MASS /BODY FAT INDICES (% Pre-Post)			
BASELINE PREDICTORS		∆ TLM	∆ ASM	∆ ASMI	∆ TLM/BW	∆ TLM/BMI	∆ ASM/BW	∆ ASM/BMI	∆ TLM/FM	∆ TLM/% FM	∆ ASM/FM	∆ ASM/% FM
***Obesity***												
	**1. BMI**	r = −0.23	**r = −0.36**	**r = −0.38**	**r = −0.67**	**r = −0.56**	r = −0.33	r = −0.29	r = −0.06	r = −0.11	r = −0.15	r = −0.22
		*p* = 0.212	***p* = 0.043**	***p* = 0.030**	***p* = 0.000**	***p* = 0.001**	*p* = 0.067	*p* = 0.106	*p* = 0.747	*p* = 0.536	*p* = 0.407	*p* = 0.234
	**2. % Body Fat**	r = −0.11	r = −0.32	**r = −0.359**	**r = −0.99**	**r = −0.82**	r = −0.27	r = −0.25	r = 0.04	r = 0.01	r = −0.08	r = −0.13
		*p* = 0.555	*p* = 0.076	***p* = 0.044**	***p* = 0.000**	***p* = 0.000**	*p* = 0.136	*p* = 0.171	*p* = 0.812	*p* = 0.965	*p* = 0.682	*p* = 0.489
	**3. Waist-to-Hip Ratio**	r = −0.19	r = −0.31	**r = −0.35**	**r = −0.48**	r = −0.30	**r = −0.42**	r = −0.27	r = −0.06	r = −0.11	r = −0.14	r = −0.20
		*p* = 0.291	*p* = 0.085	***p* = 0.050**	***p* = 0.005**	*p* = 0.100	***p* = 0.016**	*p* = 0.133	*p* = 0.759	*p* = 0.554	*p* = 0.461	*p* = 0.281
***CVD Risk***												
	**4. Systolic BP**	r = 0.09	r = 0.06	r = 0.06	r = −0.04	r = 0.10	r = 0.08	r = 0.04	r = −0.08	r = −0.05	r = −0.08	r = −0.07
		*p* = 0.635	*p* = 0.738	*p* = 0.753	*p* = 0.841	*p* = 0.573	*p* = 0.663	*p* = 0.806	*p* = 0.680	*p* = 0.765	*p* = 0.648	*p* = 0.721
	**5. Diastolic BP**	r = −0.03	r = −0.18	r = −0.20	r = −0.04	r = 0.16	r = −0.17	r = −0.19	r = −0.12	r = −0.11	r = −0.20	r = −0.21
		*p* = 0.891	*p* = 0.329	*p* = 0.281	*p* = 0.810	*p* = 0.374	*p* = 0.362	*p* = 0.294	*p* = 0.522	*p* = 0.549	*p* = 0.261	*p* = 0.252
***Dyslipidemia***												
	**6. Triglycerides**	r = −0.33	r = −0.25	r = −0.30	**r = −0.39**	**r = −0.46**	r = −0.10	r = −0.09	r = −0.14	r = −0.28	r = −0.13	r = −0.26
		*p* = 0.72	*p* = 0.180	*p* = 0.098	***p* = 0.029**	***p* = 0.009**	*p* = 0.580	*p* = 0.637	*p* = 0.458	*p* = 0.127	*p* = 0.487	*p* = 0.151
	**7. HDL**	r = 0.33	r = 0.27	r = 0.33	**r = 0.72**	**r = 0.55**	r = 0.20	r = 0.12	r = 0.16	r = 0.24	r = 0.16	r = 0.24
		*p* = 0.067	*p* = 0.144	*p* = 0.069	***p* = 0.000**	***p* = 0.001**	*p* = 0.284	*p* = 0.505	*p* = 0.398	*p* = 0.186	*p* = 0.404	*p* = 0.194
***Dysglycemia***												
	**8. HbA1c**	r = −0.28	r = −0.17	r = −0.20	r = −0.23	r = 0.03	r = −0.13	r = −0.11	r = −0.06	r = −0.19	r = −0.03	r = −0.16
		*p* = 0.125	*p* = 0.348	*p* = 0.289	*p* = 0.206	*p* = 0.869	*p* = 0.495	*p* = 0.550	*p* = 0.732	*p* = 0.305	*p* = 0.861	*p* = 0.400
***Inflammation***												
	**9. CRP**	r = −0.23	r = −0.21	r = −0.19	r = 0.07	r = 0.03	r = −0.25	r = −0.21	r = −0.19	r = −0.25	r = −0.19	r = −0.26
		*p =* 0.214	*p* = 0.259	*p* = 0.311	*p* = 0.714	*p* = 0.869	*p* = 0.178	*p* = 0.263	*p* = 0.327	*p* = 0.175	*p* = 0.311	*p* = 0.165
***Global MetS Risk Index***												
	**10. # Risk Factors**	**r = −0.45**	**r = −0.40**	**r = −0.45**	**r = −0.53**	**r = −0.37**	r = −0.30	r = −0.25	r = −0.26	**r = −0.40**	r = −0.27	**r = −0.42**
		***p* = 0.010**	***p* = 0.023**	***p* = 0.011**	***p* = 0.002**	***p* = 0.037**	*p* = 0.097	*p* = 0.159	*p* = 0.155	***p* = 0.022**	*p* = 0.134	***p* = 0.016**

**Table 3:** Participant descriptives.

	Obese/MetS			
	PLA		M5	
Descriptive Data	Pre	Post	Pre	Post
**Chronological**				
Age (years)	75 ± 1.3	-	75 ± 1.8	-
**Anthropometric**				
Height (cm)	174.2 ± 2.3	-	174.3 ± 1.1	-
BW (kg)	**91.9 ± 3.5**	**93.9 ± 3.9 ^‡^**	93.8 ± 3.4	94.6 ± 3.6
∆%	**2.2%**		0.9%	
BMI (kg/m^2^)	**30.2 ± 0.71**	**30.8 ± 0.64 ^‡^**	30.8 ± 1.06	31.1 ± 1.16
∆%	**2.0%**		1.0%	
WHR (waist/hip; cm/cm)	1.07 ± 0.03	1.05 ± 0.03	1.04 ± 0.01	1.02 ± 0.01
∆%	−1.9%		−1.9%	
**Vital Signs**				
HR (bpm)	**69.8 ± 1.4**	**74.9 ± 3.0 ^‡^**	69.5 ± 3.9	69.5 ± 4.8
∆%	**7.3%**		0.0%	
SBP (mmHg)	132 ± 3	137 ± 5	135 ± 5	140 ± 6
∆%	3.8%		3.7%	
DPB (mmHg)	**78.3 ± 1.5**	**76.9 ± 2.6**	**73.7 ± 2.1**	**78.8 ± 2.8 ^#^**
∆%	**−1.8% ^†^**		**6.9% ^†^**	
**Metabolic Syndrome and Sarcopenic Obesity**				
Global MetS Risk Index (0 min–9 max)	4.5 ± 0.38	4.88 ± 0.35	5.00 ± 0.21	4.83 ± 0.34
∆%	8.4%		−3.4%	
Sarcopenic Obesity Risk Rank (1 min–20 max)	**7.43 ± 1.49**	**9.14 ± 1.53 ^##^**	**10.33 ± 1.23**	**9.44 ± 1.22**
∆%	31.1% **		−7.4% **	
**Medical History**				
Diagnosed comorbidities (#)	3.25 ± 0.31	-	3.17 ± 0.51	-
Prescription medications (#)	3.63 ± 0.8	-	5.33 ± 1.0	-

Between-group differences in the treatment response (i.e., Δ% changes) were analyzed by independent *t*-tests (** *p* ≤ 0.01; ^†^ *p* > 0.05 < 0.100). Within-group differences in pre-post intervention results were analyzed by paired *t*-tests (^#^ *p* ≤ 0.05; ^##^ *p* ≤ 0.01; ^‡^ *p* > 0.05 < 0.100). For clarity, outcomes and *p*-values that are borderline or statistically significant are in bold. The Global MetS Risk Index and Sarcopenic Obesity Risk Rank are defined in Section 2.11. Sample size for descriptive data *n* = 18–20 (PLA; *n* = 7–8, M5; *n* = 11–12).

**Table 4:** Physical activity (PA; steps), home-based resistance exercise (HBRE) progression, and exercise/PA compliance.

	Obese/MetS			
	PLA		M5	
Physical Activity and Exercise	Pre	Post	Pre	Post
**PA**				
Daily activity (steps/day)	**6149 ± 1078**	**4735 ± 668 ^#^**	**4369 ± 484**	**4921 ± 568**
∆%	−23% *		13% *	
**HBRE**				
Elastic band resistance (kg)	**2.19 ± 0.22**	**2.98 ± 0.17 ^#^**	**1.96 ± 0.14**	**2.55 ± 0.14 ^##^**
∆%	**36%**		**30%**	
**Compliance**				
Step goals	-	7857/d (60%)	-	7857/d (63%)
HBRE (%)	-	89 ± 12	-.	76 ± 7

Between-group differences (i.e., Δ% changes) were analyzed by independent *t*-tests (* *p* ≤ 0.05). Within-group differences in pre-post intervention results were analyzed by paired *t*-tests (^#^ *p* ≤ 0.05; ^##^ *p* ≤ 0.01). For clarity, outcomes and *p*-values that are borderline or statistically significant are in bold. Sample size for physical activity and exercise outcomes *n* = 19 (PLA; *n* = 8, M5; *n* = 11).

**Table 5:** Supplement ingredient list and compliance.

	PLA	M5
Ingredients	Per Serving	Per Serving
**Macronutrients**	**Collagen peptides**	**Muscle 5**
Calories (kcal)	272	272
Protein (g)	40	40
Sucrose (g)	6.4	6.4
Fat (g)	0.5	0.5
**Actives**	**Collagen peptides**	**Muscle 5**
Whey Protein Isolate (WPI 895; g)	-	24
Milk Protein Isolate (MPI 4900; g)	-	16
Bovine collagen (Peptiplus; g)	40	-
Creatine monohydrate (g)	-	3
Vitamin D_3_ (UI)	0.0	1000
Calcium (mg)	0.0	416
**Oils and fatty acids (FAs)**	**Safflower oil (two tsp.)**	**Fish oil (two tsp.)**
Total oil contents (mono-, di-, triglycerides etc.) (g)	8.6	8.6
Long-chain omega-3 PUFAs (C20–C22; g)	0.0	2.46
Eicosapentaenoic acid (C20; EPA) (g)	0.0	1.51
Docosahexaenoic acid (C22; DHA) (g)	0.0	0.95
Other FAs (%)	100% (linoleic > oleic > palmitic > stearic)	71%
Vitamin E (d-alpha tocopherol) (g)	0.1	0.08
**Compliance**	**PLA**	**M5**
Supplement intake (%)	95.40 ± 1.9	89.29 ± 5.0

**Table 6:** Macronutrient analyses 72-h dietary recall (food only).

	Obese/MetS			
	PLA		M5	
Dietary Intake	Pre	Post	Pre	Post
**Macronutrients**				
Calories (kcal/d)	**1602 ± 119**	**1928 ± 73 ^#^**	**2027 ± 169**	**1751 ± 140 ^#^**
∆%	20% **		−14% **	
Protein (g/d)	**77 ± 10**	**92 ± 5 ^‡^**	**84 ± 8**	**80 ± 6**
∆%	19% *		−5% *	
Protein (g/kg BW/d)	**0.84 ± 0.08**	**1.00 ± 0.08 ^‡^**	**0.92 ± 0.10**	**0.87 ± 0.08**
∆%	19% *		−5% *	
Fat (g/d)	**71 ± 9**	**80 ± 6**	**79 ± 8**	**73 ± 11**
∆%	**13% ^†^**		**−8% ^†^**	
Carbohydrates (g/d)	**154 ± 12**	**205 ± 11 ^##^**	**221 ± 27**	**184 ± 23 ^#^**
∆%	33% **		−17% **	

Between-group differences (i.e., Δ% changes) were analyzed by independent *t*-tests (* *p* ≤ 0.05; ** *p* ≤ 0.01; ^†^ *p* > 0.05 < 0.100). Within-group differences in pre-post intervention results were analyzed by paired *t*-tests (^#^ *p* ≤ 0.05; ^##^ *p* ≤ 0.01; ^‡^ *p* > 0.05 < 0.100). For clarity, outcomes and *p*-values that are borderline or statistically significant are in bold. Sample size for macronutrient outcomes *n* = 18 (PLA; *n* = 8, M5; *n* = 10).

**Table 7:** Blood chemistry.

	Obese/MetS			
	PLA		M5	
Blood Chemistry	Pre	Post	Pre	Post
**Lipid Profile**				
Cholesterol (mmol/L)	3.39 ± 0.31	3.53 ± 0.36	4.38 ± 0.38	4.17 ± 0.41
∆%	4.1%		−4.8%	
Triglycerides (mmol/L)	1.26 ± 0.20	1.27 ± 0.15	1.74 ± 0.24	1.60 ± 0.19
∆%	0.8%		−8.0%	
HDL-Cholesterol (mmol/L)	1.09 ± 0.10	1.12 ± 0.11	1.01 ± 0.05	1.00 ± 0.05
∆%	2.8%		−1.0%	
LDL-Cholesterol (mmol/L)	1.72 ± 0.26	1.83 ± 0.32	2.57 ± 0.38	2.43 ± 0.39
∆%	6.4%		−5.4%	
Non-HDL-Cholesterol (mmol/L)	2.30 ± 0.26	2.42 ± 0.28	3.36 ± 0.36	3.17 ± 0.37
∆%	5.2%		−5.7%	
TC/HDL-C	3.15 ± 0.28	3.19 ± 0.22	4.33 ± 0.31	4.13 ± 0.28
∆%	1.3%		−4.6%	
**Hemoglobin A1C Test**				
HbA1c (%)	6.09 ± 0.38	6.14 ± 0.32	6.12 ± 0.24	6.17 ± 0.25
∆%	0.8%		0.8%	
**C-reactive Protein Test**				
CRP (mg/L)	1.66 ± 0.50	1.84 ± 0.46	2.34 ± 0.72	2.44 ± 0.74
∆%	10.8%		4.3%	
**Bone Markers**				
P1NP (pg/mL)	283 ± 108	267 ± 87	**163 ± 28**	**228 ± 40 ^#^**
∆%	−5.6%		**39.9%**	
CTX (pg/mL)	193 ± 14	202 ± 7.5	**197 ± 11**	**206 ± 11 ^‡^**
∆%	4.6%		**4.5%**	
P1NP/CTX ratio	1.56 ± 0.65	1.31 ± 0.39	**0.85 ± 0.15**	**1.26 ± 0.25 ^‡^**
∆%	−16%		**48.2%**	

Between-group differences in the treatment response (i.e., Δ% changes) were analyzed by independent *t*-tests (no significances detected). Within-group differences in pre-post intervention results were analyzed by paired *t*-tests (^#^ *p* ≤ 0.05; ^‡^ *p* > 0.05 < 0.100). For clarity, outcomes and *p*-values that are borderline or statistically significant are in bold. Sample size for metabolic risk markers *n* = 15–19 (PLA; *n* = 5–8, M5; *n* = 10–11) and bone turnover markers *n* = 9–11 (PLA; *n* = 4, M5; *n* = 5–7).

**Table 8:** Body composition.

	Obese/MetS			
	PLA		M5	
DEXA	Pre	Post	Pre	Post
**Lean Mass**				
TLM (kg)	56.6 ± 2.2	56.3 ± 2.2	57.1 ± 2.1	57.8 ± 2.0
∆%	−0.5%		1.2%	
ASM (kg)	24.5 ± 0.8	24.3 ± 0.8	**25.1 ± 1.0**	**25.6 ± 1.0 ^‡^**
∆%	−0.8%		**2.0%**	
**Fat Mass**				
Total Fat Mass (FM; kg)	**32.1 ± 1.9**	**33.6 ± 2.1 ^#^**	**33.3 ± 2.3**	**33.0 ± 2.5**
∆%	**4.7% ^†^**		**−0.9% ^†^**	
Body Fat (BF; %)	**36.1 ± 1.1**	**37.2 ± 1.1 ^#^**	**36.6 ± 1.7**	**35.8 ± 1.6 ^#^**
∆%	3.0% **		−2.2% **	
**Body Composition Indices**				
TLM/FM	**1.79 ± 0.08**	**1.70 ± 0.07 ^#^**	**1.81 ± 0.15**	**1.86 ± 0.15**
∆%	−5.0% *		2.8% *	
ASM/FM	**0.78 ± 0.04**	**0.74 ± 0.04 ^#^**	**0.79 ± 0.06**	**0.82 ± 0.07**
∆%	−5.1% **		3.8% **	
TLM/%BF	**1.58 ± 0.08**	**1.52 ± 0.07**	**1.62 ± 0.11**	**1.67 ± 0.11 ^#^**
∆%	−3.8% *		3.1% *	
ASM/%BF	**0.68 ± 0.03**	**0.66 ± 0.03**	**0.71 ± 0.05**	**0.74 ± 0.05 ^#^**
∆%	−2.9% **		4.2% **	
**Allometric Lean Mass**				
TLM/BMI	**1.87 ± 0.05**	**1.82 ± 0.05 ^#^**	**1.86 ± 0.06**	**1.87 ± 0.06**
∆%	−2.7% *		0.5% *	
TLM/BW	**0.62 ± 0.01**	**0.60 ± 0.01 ^#^**	**0.61 ± 0.02**	**0.61 ± 0.02**
∆%	−3.2% *		0.0% *	
ASM/h^2^ (ASMI; kg/m^2^)	8.08 ± 0.25	8.02 ± 0.21	**8.25 ± 0.30**	**8.41 ± 0.32 ^‡^**
∆%	−0.7%		**1.9%**	
ASM/BMI (kg/[kg/m^2^])	**0.81 ± 0.02**	**0.79 ± 0.03 ^‡^**	**0.82 ± 0.02**	**0.83 ± 0.03**
∆%	−2.5% *		1.2% *	
ASM/BW	**0.27 ± 0.01**	**0.26 ± 0.01 ^#^**	**0.27 ± 0.01**	**0.27 ± 0.01**
∆%	−3.7% *		0.0% *	
**Bone Mass**				
Bone Mineral Density (g/cm^2^)	1.23 ± 0.03	1.24 ± 0.03	1.21 ± 0.03	1.21 ± 0.03
∆%	0.8%		0.0%	

Between-group differences in the treatment response (i.e., Δ% changes) were analyzed by independent *t*-tests (* *p* ≤ 0.05; ** *p* ≤ 0.01; ^†^ *p* > 0.05 < 0.100). Within-group differences in pre-post intervention results were analyzed by paired *t*-tests (^#^ *p* ≤ 0.05; ^‡^ *p* > 0.05 < 0.100). For clarity, outcomes and *p*-values that are borderline or statistically significant are in bold. Sample size for analyses *n* = 20 (PLA; *n* = 8, M5; *n* = 12). Sample size for body composition outcomes *n* = 20 (PLA; *n* = 8, M5; *n* = 12).

**Table 9:** Strength and Muscle Quality.

	Obese/MetS			
	PLA		M5	
Strength	Pre	Post	Pre	Post
**Lower Body**				
Leg Press 1RM (kg)	144 ± 16	142 ± 16	**120 ± 9**	**140 ± 9 ^#^**
∆%	−1.4%		**17% ^†^**	
Isometric Knee Extension (Nm)	189 ± 13	194 ± 16	170 ± 11	183 ± 11
∆%	2.6%		7.6%	
**Upper Body**				
Hand Grip (kg)	40.7 ± 2.8	45.3 ± 3.0	**39.1 ± 1.8**	**42.7 ± 1.7 ^#^**
∆%	11.3%		**9.2%**	
**Muscle Quality**				
MQ Index	47.4 ± 2.7	52.0 ± 3.3	**44.9 ± 2.0**	**49.0 ± 1.8 ^##^**
∆%	9.7%		**9.1%**	

Between-group differences in the treatment response (i.e., Δ% changes) were analyzed by independent *t*-tests (^†^ *p* > 0.05 < 0.100). Within-group differences in pre-post intervention results were analyzed by paired *t*-tests (^#^ *p* ≤ 0.05; ^##^ *p* ≤ 0.01). For clarity, outcomes and *p*-values that are borderline or statistically significant are in bold. Sample size for strength outcomes *n* = 19 (PLA; *n* = 7, M5; *n* = 12).

**Table 10:** Performance.

	Obese/MetS			
	PLA		M5	
Performance	Pre	Post	Pre	Post
**Walk Speed**				
4-M (m/s)	1.01 ± 0.06	1.01 ± 0.05	0.87 ± 0.06	0.90 ± 0.06
∆%	0.0%		3.4%	
**Functional Mobility**				
Timed 5x Sit to Stand (5XSTS; s)	11.0 ± 1.3	10.8 ± 1.2	12.5 ± 0.5	11.8 ± 0.9
∆%	−1.8%		−5.6%	
Timed 4-Step Stair Climb (4SSC; s)	2.64 ± 0.18	2.67 ± 0.12	**3.25 ± 0.20**	**2.91 ± 0.13 ^‡^**
∆%	1.1%		**−10.5%**	
**Short Physical Performance Battery**				
SPPB Score (max 12)	11.0 ± 0.69	10.9 ± 0.70	10.3 ± 0.35	10.4 ± 0.53
∆%	−0.9%		1.0%	

Between-group differences in the treatment response (i.e., Δ% changes) were analyzed by independent *t*-tests (no significance detected). Within-group differences in pre-post intervention results were analyzed by paired *t*-tests (^‡^ *p* > 0.05 < 0.100). For clarity, outcomes and *p*-values that are borderline or statistically significant are in bold. Sample size for performance outcomes *n* = 19 (PLA; *n* = 7, M5; *n* = 12).

## Data Availability

The data presented in this study are available on request from the corresponding author due to privacy.

## Supplementary Materials

The following supporting information can be downloaded at: https://www.mdpi.com/article/10.3390/nu16244407/s1, Table S1: Prediction Model 1 (strength and function). Table S2: Prediction Model 2 (strength and function). Table S3: Collinearity Model 1. Table S4: Collinearity Model 2. Table S5: Supplement Amino Acid Profiles. Table S6: Kidney and Liver Markers. Between-group differences in the adaptive response (i.e., Δ% changes) were analyzed by independent *t*-tests (* *p* ≤ 0.05). Within-group differences in pre-post intervention results were analyzed by paired *t*-tests (^#^ *p* ≤ 0.05). Sample size for kidney and liver outcomes *n* = 19 (PLA; *n* = 8, M5; *n* = 11).
