# Supplementary material for: Obesity and Metabolic Disease Impair the Anabolic Response to Protein Supplementation and Resistance Exercise: A Retrospective Analysis of a Randomized Clinical Trial with Implications for Aging, Sarcopenic Obesity, and Weight Management
Source: Nutrients. 2024 Dec 23;16(24):4407. doi: 10.3390/nu16244407 (PMC11677392; doi:10.3390/nu16244407)
Supplement: Supplementary file 1 [file nutrients-16-04407-s001.zip › TABLE S1. PREDICT MOD 1 (STRENGTH FUNCTION).pdf]

Table S1. Prediction Model 1 (strength & function).

PREDICTORS (Pre-intervention)	PREDICTION MODEL 1<br>Δ STRENGTH (% pre-post intervention)<br>Δ Grip Strength	 <br>Δ Leg Press	 Δ PERFORMANCE (% pre-post intervention)<br>Δ SPPB	 <br>Δ 5x Sit-to-Stand
1. Age (years)	r = -0.38<br>*p* = 0.052	r = -0.11<br>*p* = 0.555	r = 0.24<br>*p* = 0.195	r = -0.15<br>*p* = 0.434
2. Obesity (BMI)	r = 0.30<br>*p* = 0.103	r = -0.03<br>*p* = 0.880	***r = -0.40***<br>***p = 0.028***	***r = 0.52***<br>***p = 0.004***
3. PA (steps/day)	r = -0.06<br>*p* = 0.768	r = -0.17<br>*p* = 0.384	r = -0.17<br>*p* = 0.377	r = 0.06<br>*p* = 0.749
4. Kidney Function				
**a. eGFR (mL/min/1.73 m^2)**	r = 0.10<br>*p* = 0.597	r = 0.16<br>*p* = 0.420	r = -0.05<br>*p* = 0.797	r = -0.13<br>*p* = 0.499
**b. Creatinine (µmol/L)**	r = -0.04<br>*p* = 0.829	r = -0.17<br>*p* = 0.374	r = -0.01<br>*p* = 0.945	r = 0.15<br>*p* = 0.447
6. Protein Intake (g/kgBW/d)	***r = -0.41***<br>***p = 0.023***	r = -0.23<br>*p* = 0.228	r = 0.11<br>*p* = 0.579	r = -0.33<br>*p* = 0.078
7. Exercise Adherence (%)	r = 0.11<br>*p* = 0.570	r = 0.04<br>*p* = 0.845	r = 0.31<br>*p* = 0.103	r = -0.10<br>*p* = 0.621
8. Supplement Adherence (%)	r = 0.21<br>*p* = 0.282	r = -0.01<br>*p* = 0.940	r = -0.30<br>*p* = 0.118	r = 0.29<br>*p* = 0.138
