# Supplementary material for: Obesity and Metabolic Disease Impair the Anabolic Response to Protein Supplementation and Resistance Exercise: A Retrospective Analysis of a Randomized Clinical Trial with Implications for Aging, Sarcopenic Obesity, and Weight Management
Source: Nutrients. 2024 Dec 23;16(24):4407. doi: 10.3390/nu16244407 (PMC11677392; doi:10.3390/nu16244407)
Supplement: Supplementary file 1 [file nutrients-16-04407-s001.zip › TABLE S2. PREDICT MOD 2 (STRENGTH FUNCTION).pdf]

Table S2. Prediction Model 2 (strength & function).

	PREDICTION MODEL 2			
	**Δ STRENGTH (% pre-post intervention)**		**Δ PERFORMANCE (% pre-post intervention)**	
**PREDICTORS (Pre-intervention)**	**Δ Grip Strength**	**Δ Leg Press**	**Δ SPPB**	**Δ 5x Sit-to-Stand**
Obesity				
**1. BMI**	r = 0.30<br>*p* = 0.103	r = -0.03<br>*p* = 0.880	***r = -0.40***<br>***p = 0.028***	***r = 0.52***<br>***p = 0.004***
**2. % Body Fat**	***r = 0.37***<br>***p = 0.046***	r = 0.20<br>*p* = 0.291	r = -0.17<br>*p* = 0.362	r = 0.35<br>*p* = 0.060
**3. Waist-to-Hip Ratio**	r = 0.31<br>*p* = 0.091	r = 0.07<br>*p* = 0.705	***r = -0.40***<br>***p = 0.028***	***r = 0.52***<br>***p = 0.004***
CVD Risk				
**4. Systolic BP**	r = 0.12<br>*p* = 0.537	r = 0.18<br>*p* = 0.332	r = 0.26<br>*p* = 0.162	r = -0.26<br>*p* = 0.160
**5. Diastolic BP**	r = 0.10<br>*p* = 0.613	r = 0.02<br>*p* = 0.915	r = 0.03<br>*p* = 0.863	r = -0.13<br>*p* = 0.504
Dyslipidemia				
**6. Triglycerides**	r = 0.11<br>*p* = 0.586	r = -0.15<br>*p* = 0.451	r = -0.21<br>*p* = 0.275	r = 0.03<br>*p* = 0.877
**7. HDL**	r = -0.30<br>*p* = 0.116	r = 0.03<br>*p* = 0.892	r = 0.20<br>*p* = 0.301	r = -0.28<br>*p* = 0.148
Dysglycemia				
**8. HbA1c**	r = 0.22<br>*p* = 0.257	r = -0.10<br>*p* = 0.615	r = -0.00<br>*p* = 0.996	r = 0.19<br>*p* = 0.321
Inflammation				
**9. CRP**	r = -0.16<br>*p* = 0.404	r = -0.36<br>*p* = 0.059	r = -0.29<br>*p* = 0.141	r = 0.10<br>*p* = 0.611
Global MetS Risk Index				
**10. Risk Factors (9 max)**	r = 0.19<br>*p* = 0.309	r = 0.07<br>*p* = 0.722	r = -0.20<br>*p* = 0.302	r = 0.184<br>*p* = 0.323
