# Supplementary material for: Obesity and Metabolic Disease Impair the Anabolic Response to Protein Supplementation and Resistance Exercise: A Retrospective Analysis of a Randomized Clinical Trial with Implications for Aging, Sarcopenic Obesity, and Weight Management
Source: Nutrients. 2024 Dec 23;16(24):4407. doi: 10.3390/nu16244407 (PMC11677392; doi:10.3390/nu16244407)
Supplement: Supplementary file 1 [file nutrients-16-04407-s001.zip › TABLE S3. COLLINEARITY (MODEL 1).pdf]

**Table S3. Collinearity Model 1.**

| | Age (years) | Obesity (BMI) | PA (steps/day) | eGFR (mL/min/1.73 $m^2$) | Creatinine (μmol/L) | Protein (g/kgBW/d) | Exercise Adherence (%) | Supplement Adherence (%) |
|---|---|---|---|---|---|---|---|---|
| **Age (years)** | --- | r = -0.13<br>*p* = 0.512 | r = -0.24<br>*p* = 0.226 | ***r = -0.57***<br>***p = 0.002*** | ***r = 0.48***<br>***p = 0.13*** | r = 0.17<br>*p* = 0.393 | r = 0.02<br>*p* = 0.918 | ***r = -0.50***<br>***p = 0.006*** |
| **Obesity (BMI)** | r = -0.13<br>*p* = 0.512 | --- | r = 0.01<br>*p* = 0.952 | r = 0.17<br>*p* = 0.403 | r = -0.18<br>*p* = 0.378 | ***r = -0.53***<br>***p = 0.005*** | r = -0.37<br>*p* = 0.065 | r = 0.22<br>*p* = 0.280 |
| **PA (steps/day)** | r = -0.24<br>*p* = 0.226 | r = 0.01<br>*p* = 0.952 | --- | r = 0.27<br>*p* = 0.170 | r = -0.18<br>*p* = 0.378 | r = 0.05<br>*p* = 0.505 | r = -0.03<br>*p* = 0.892 | r = 0.26<br>*p* = 0.189 |
| **eGFR (mL/min/1.73 $m^2$)** | ***r = -0.57***<br>***p = 0.003*** | r = 0.17<br>*p* = 0.403 | r = 0.27<br>*p* = 0.170 | --- | ***r = -0.98***<br>***p = 0.000*** | r = -0.14<br>*p* = 0.472 | r = -0.01<br>*p* = 0.968 | ***r = 0.45***<br>***p = 0.015*** |
| **Creatinine (μmol/L)** | ***r = 0.48***<br>***p = 0.013*** | r = -0.18<br>*p* = 0.378 | r = -0.30<br>*p* = 0.138 | ***r = -0.98***<br>***p = 0.000*** | --- | r = 0.15<br>*p* = 0.472 | r = 0.04<br>*p* = 0.864 | ***r = -0.50***<br>***p = 0.010*** |
| **Protein (g/kgBW/d)** | r = 0.17<br>*p* = 0.393 | ***r = -0.53***<br>***p = 0.005*** | r = 0.05<br>*p* = 0.808 | r = -0.14<br>*p* = 0.472 | ***r = 0.48***<br>***p = 0.013*** | --- | r = 0.03<br>*p* = 0.884 | r = -0.10<br>*p* = 0.628 |
| **Exercise Adherence (%)** | r = 0.02<br>*p* = 0.918 | r = -0.37<br>*p* = 0.065 | r = -0.03<br>*p* = 0.892 | r = -0.01<br>*p* = 0.968 | r = 0.04<br>*p* = 0.864 | r = 0.03<br>*p* = 0.884 | --- | r = -0.05<br>*p* = 0.826 |
| **Supplement Adherence (%)** | ***r = -0.50***<br>***p = 0.006*** | r = 0.22<br>*p* = 0.280 | r = 0.26<br>*p* = 0.189 | ***r = 0.45***<br>***p = 0.015*** | ***r = -0.50***<br>***p = 0.010*** | r = -0.10<br>*p* = 0.628 | r = -0.03<br>*p* = 0.887 | --- |
