# Supplementary material for: Obesity and Metabolic Disease Impair the Anabolic Response to Protein Supplementation and Resistance Exercise: A Retrospective Analysis of a Randomized Clinical Trial with Implications for Aging, Sarcopenic Obesity, and Weight Management
Source: Nutrients. 2024 Dec 23;16(24):4407. doi: 10.3390/nu16244407 (PMC11677392; doi:10.3390/nu16244407)
Supplement: Supplementary file 1 [file nutrients-16-04407-s001.zip › TABLE S4. COLLINEARITY (MODEL 2).pdf]

Table S4. Collinearity Model 2.

	BMI	% Body Fat	Waist-to-Hip Ratio	Systolic BP	Diastolic BP	Triglycerides	HDL	HbA1c	CRP	Global MetS Risk Index
**BMI**	---	***r* = 0.75**<br>***p* = 0.000**	***r* = 0.58**<br>***p* = 0.002**	r = -0.30<br>*p* = 0.143	r = -0.14<br>*p* = 0.501	***r* = 0.55**<br>***p* = 0.003**	***r* = -0.76**<br>***p* = 0.000**	r = 0.40<br>*p* = 0.045	r = 0.05<br>*p* = 0.816	***r* = 0.57**<br>***p* = 0.002**
**% Body Fat**	***r* = 0.75**<br>***p* = 0.000**	---	***r* = 0.54**<br>***p* = 0.005**	r = -0.18<br>*p* = 0.377	r = -0.17<br>*p* = 0.420	r = 0.49<br>*p* = 0.011	***r* = -0.80**<br>***p* = 0.000**	r = 0.35<br>*p* = 0.076	r = 0.04<br>*p* = 0.833	***r* = 0.59**<br>***p* = 0.002**
**Waist-to-Hip Ratio**	***r* = 0.58**<br>***p* = 0.002**	***r* = 0.54**<br>***p* = 0.005**	---	r = -0.26<br>*p* = 0.206	r = 0.05<br>*p* = 0.796	r = 0.24<br>*p* = 0.231	***r* = -0.49**<br>***p* = 0.011**	***r* = 0.50**<br>***p* = 0.010**	r = 0.07<br>*p* = 0.750	***r* = 0.54**<br>***p* = 0.004**
**Systolic BP**	r = -0.30<br>*p* = 0.143	r = -0.18<br>*p* = 0.377	r = -0.26<br>*p* = 0.206	---	***r* = 0.48**<br>***p* = 0.012**	r = -0.14<br>*p* = 0.481	r = 0.17<br>*p* = 0.420	r = 0.04<br>*p* = 0.863	r = 0.08<br>*p* = 0.707	r = 0.23<br>*p* = 0.259
**Diastolic BP**	r = -0.14<br>*p* = 0.501	r = -0.17<br>*p* = 0.420	r = 0.05<br>*p* = 0.796	***r* = 0.48**<br>***p* = 0.012**	---	r = -0.16<br>*p* = 0.431	r = 0.20<br>*p* = 0.319	r = -0.05<br>*p* = 0.806	r = -0.13<br>*p* = 0.513	r = -0.00<br>*p* = 0.982
**Triglycerides**	***r* = 0.55**<br>***p* = 0.003**	***r* = 0.49**<br>***p* = 0.011**	r = 0.24<br>*p* = 0.231	r = -0.14<br>*p* = 0.481	r = -0.16<br>*p* = 0.431	---	***r* = -0.66**<br>***p* = 0.000**	r = 0.30<br>*p* = 0.140	r = -0.24<br>*p* = 0.247	***r* = 0.66**<br>***p* = 0.000**
**HDL**	***r* = -0.77**<br>***p* = 0.000**	***r* = -0.80**<br>***p* = 0.000**	***r* = -0.49**<br>***p* = 0.011**	r = 0.17<br>*p* = 0.420	r = 0.20<br>*p* = 0.319	***r* = -0.66**<br>***p* = 0.000**	---	r = -0.41<br>*p* = 0.036	r = 0.07<br>*p* = 0.723	***r* = -0.68**<br>***p* = 0.000**
**HbA1c**	***r* = 0.40**<br>***p* = 0.045**	r = 0.35<br>*p* = 0.076	***r* = 0.50**<br>***p* = 0.010**	r = -0.04<br>*p* = 0.863	r = -0.05<br>*p* = 0.806	r = 0.30<br>*p* = 0.140	***r* = -0.41**<br>***p* = 0.036**	---	r = -0.19<br>*p* = 0.356	***r* = 0.49**<br>***p* = 0.011**
**CRP**	r = 0.05<br>*p* = 0.816	r = 0.04<br>*p* = 0.833	r = 0.07<br>*p* = 0.750	r = 0.08<br>*p* = 0.707	r = -0.13<br>*p* = 0.513	r = -0.24<br>*p* = 0.247	r = 0.07<br>*p* = 0.723	r = -0.19<br>*p* = 0.356	---	r = 0.11<br>*p* = 0.606
**Global MetS Risk Index**	***r* = 0.57**<br>***p* = 0.002**	***r* = 0.59**<br>***p* = 0.002**	***r* = 0.54**<br>***p* = 0.004**	r = 0.23<br>*p* = 0.259	r = -0.00<br>*p* = 0.982	***r* = 0.66**<br>***p* = 0.000**	***r* = -0.68**<br>***p* = 0.000**	***r* = 0.49**<br>***p* = 0.011**	r = 0.11<br>*p* = 0.606	---
