# Supplementary material for: Obesity and Metabolic Disease Impair the Anabolic Response to Protein Supplementation and Resistance Exercise: A Retrospective Analysis of a Randomized Clinical Trial with Implications for Aging, Sarcopenic Obesity, and Weight Management
Source: Nutrients. 2024 Dec 23;16(24):4407. doi: 10.3390/nu16244407 (PMC11677392; doi:10.3390/nu16244407)
Supplement: Supplementary file 1 [file nutrients-16-04407-s001.zip › TABLE S5. AMINO PROFILES.pdf]

**Table S5. Supplement Amino Acid Profiles.**

| | PLA (Collagen + Safflower Oil) | | Muscle 5 (M5 + Fish Oil) | | |
|---|---|---|---|---|---|
| | Peptiplus* | | MPI 4900* | WPI 895* | |
| **Amino Acid Breakdown Per Serving** | | | | | |
| **Essential Amino Acids** | **g** | **% total AAs** | **g** | **g** | **% total AAs** |
| Histidine | 0.40 | 1.0 | 0.43 | 0.48 | 2.1 |
| Isoleucine | 0.56 | 1.4 | 0.85 | 1.51 | 5.4 |
| Leucine | 1.08 | 2.7 | 1.54 | 3.43 | 11.4 |
| Lysine | 1.44 | 3.6 | 1.34 | 2.69 | 9.2 |
| Methionine | 0.36 | 0.9 | 0.45 | 0.58 | 2.4 |
| Phenylalanine | 0.84 | 2.1 | 0.78 | 0.91 | 3.9 |
| Threonine | 0.72 | 1.8 | 0.72 | 1.27 | 4.6 |
| Tryptophan | ND | ND | 0.22 | 0.58 | 1.8 |
| Valine | 0.96 | 2.4 | 1.02 | 1.34 | 5.4 |
| **Total** | **6.36** | **15.9** | **7.36** | **12.79** | **46.2** |
| **Non-Essential Amino Acids** | **g** | **% total AAs** | **g** | **g** | **% total AAs** |
| Alanine | 3.44 | 8.6 | 0.53 | 1.37 | 4.4 |
| Arginine | 2.92 | 7.3 | 0.59 | 0.72 | 3.0 |
| Aspartic acid | 2.32 | 5.8 | 1.25 | 3.00 | 9.7 |
| Cysteine | ND | ND | 0.19 | 0.96 | 2.6 |
| Glutamic acid | 4.08 | 10.2 | 3.46 | 4.22 | 17.6 |
| Glycine | 8.88 | 22.2 | 0.30 | 0.43 | 1.7 |
| Proline | 5.08 | 12.7 | 1.57 | 1.08 | 6.1 |
| Serine | 1.28 | 3.2 | 0.90 | 1.08 | 4.5 |
| Tyrosine | 0.32 | 0.8 | 0.85 | 1.01 | 4.3 |
| **Total** | **28.32** | **70.7** | **9.63** | **13.87** | **53.8** |
| **Collagen-Unique Amino Acids** | **g** | **% total AAs** | **g** | **g** | **% total AAs** |
| Hydroxyproline | 4.76 | 11.9 | ND | ND | ND |
| Hydroxylysine | 0.64 | 1.6 | ND | ND | ND |
| **Total** | **5.40** | **13.5** | **ND** | **ND** | **ND** |

*Based on product specification sheets (GELITA and Fonterra)
