# Supplementary material for: Obesity and Metabolic Disease Impair the Anabolic Response to Protein Supplementation and Resistance Exercise: A Retrospective Analysis of a Randomized Clinical Trial with Implications for Aging, Sarcopenic Obesity, and Weight Management
Source: Nutrients. 2024 Dec 23;16(24):4407. doi: 10.3390/nu16244407 (PMC11677392; doi:10.3390/nu16244407)
Supplement: Supplementary file 1 [file nutrients-16-04407-s001.zip › TABLE S6. KIDNEY & LIVER MARKERS.pdf]

Table S6. Kidney and Liver Markers.

	Obese/MetS			
	PLA (n = 8)		M5 (n = 12)	
**Clinical Blood Tests**	**Pre**	**Post**	**Pre**	**Post**
**Kidney Function**				
Creatinine (μmol/L)	***91 ± 4.4***	***90 ± 4.4***	***93 ± 5.1***	***102 ± 7.1*** #
Δ%	***-1.1%*** *		***9.7%*** *	
eGFR (CKD-epi) (mL/min/1.73m^2)	***71 ± 3.8***	***73 ± 3.6***	***71 ± 4.3***	***66 ± 5.0*** #
Δ%	***2.8%*** *		***-7.0%*** *	
**Liver Function**				
Bilirubin (μmol/L)	14.8 ± 1.3	13.6 ± 1.7	14.6 ± 2.4	13.6 ± 1.8
Δ%	-8.1%		6.8%	
ALT (μmol/L)	25.8 ± 3.8	25.5 ± 2.9	22.7 ± 3.7	22.7 ± 2.6
Δ%	1.1%		0.0%	
GGT (μmol/L)	25.9 ± 3.3	26.5 ± 2.8	36.4 ± 6.3	34.8 ± 5.6
Δ%	2.3%		-4.4%	

Between-group differences in the adaptive response (i.e., Δ % changes) were analyzed by independent *t*-tests (* $p \leq 0.05$). Within-group differences in pre-post intervention results were analyzed by paired *t*-tests ($^{\#}$ $p \leq 0.05$). Sample size for kidney and liver outcomes $n = 19$ (PLA; $n = 8$, M5; $n = 11$).
